# Eco-evolution in size-structured ecosystems: simulation case study of rapid morphological changes in alewife

**DOI:** 10.1186/s12862-017-0912-4

**Published:** 2017-02-27

**Authors:** Jung koo Kang, Xavier Thibert-Plante

**Affiliations:** 1 The Center for Quantitative Sciences in Biomedicine, North Carolina State University, Campus Box 8213, 308 Cox Hall, Raleigh, 27695-8212 NC USA; 2 Icelab and Department of Ecology and Environmental Science, Umeå University, Linnaeus va̋g 6, Umeå, Sweden

**Keywords:** Eco-evolution, Size-structured ecosystem, Individual-based model, Ecological power law, Contemporary evolution, Functional trait, Body size, Fish, Alewife

## Abstract

**Background:**

Over the last 300 years, interactions between alewives and zooplankton communities in several lakes in the U.S. have caused the alewives’ morphology to transition rapidly from anadromous to landlocked. Lakes with landlocked alewives contain smaller-bodied zooplankton than those without alewives. Landlocked adult alewives display smaller body sizes, narrower gapes, smaller inter-gill-raker spacings, reach maturity at an earlier age, and are less fecund than anadromous alewives. Additionally, landlocked alewives consume pelagic prey exclusively throughout their lives whereas anadromous alewives make an ontogenetic transition from pelagic to littoral prey. These rapid, well-documented changes in the alewives’ morphology provide important insights into the morphological evolution of fish.

Predicting the morphological evolution of fish is crucial for fisheries and ecosystem management, but the involvement of multiple trophic interactions make predictions difficult. To obtain an improved understanding of rapid morphological change in fish, we developed an individual-based model that simulated rapid changes in the body size and gill-raker count of a fish species in a hypothetical, size-structured prey community. Model parameter values were based mainly on data from empirical studies on alewives. We adopted a functional trait approach; consequently, the model explicitly describes the relationships between prey body size, alewife body size, and alewife gill-raker count. We sought to answer two questions: (1) How does the impact of alewife populations on prey feed back to impact alewife size and gill raker number under several alternative scenarios? (2) Will the trajectory of the landlocked alewives’ morphological evolution change after 150–300 years in freshwater?

**Results:**

Over the first 250 years, the alewives’ numbers of gill-rakers only increased when reductions in their body size substantially improved their ability to forage for small prey. Additionally, alewives’ gill-raker counts increased more rapidly as the adverse effects of narrow gill-raker spacings on foraging for large prey were made less severe. For the first 150–250 years, alewives’ growth decreased monotonically, and their gill-raker number increased monotonically. After the first 150–250 years, however, the alewives exhibited multiple evolutionary morphological trajectories in different trophic settings. In several of these settings, their evolutionary trajectories even reversed after the first 150–250 years.

**Conclusions:**

Alewives affected the abundance and morphology of their prey, which in turn changed the abundance and morphology of the alewives. Complex low-trophic-level interactions can alter the abundance and characteristics of alewives. This study suggests that the current morphology of recently (∼300 years)-landlocked alewives may not represent an evolutionarily stable state.

**Electronic supplementary material:**

The online version of this article (doi:10.1186/s12862-017-0912-4) contains supplementary material, which is available to authorized users.

## Background

### Functional trait approaches for studying rapid morphological evolution in fish

Predator-prey interactions affect contemporary evolutionary processes such as the rapid morphological changes observed in several fish species [1–4]. Predicting morphological changes in fish is crucial for fisheries and ecosystem management, but such changes are affected by a complex set of trophic interactions, which makes accurate prediction very difficult.

Functional trait approaches have been widely used to study the effect of the disturbance caused by a species on a community [5–7], but they can also be used to study how the evolution of one species in a community is affected by the other species that are present. Functional traits strongly affect organismal performance [8], so changes in species’ functional traits can affect the abundance of its predators and prey, which in turn alters its fitness landscape. For example, predator morphology can determine the size of prey that predators consume [9]. Conversely, prey size distribution can influence predator size distribution [10]. By extension, entire communities can be analyzed by studying the functional abilities of both the assemblage as a whole and the component species individually [5–7].

### Rapid changes in alewife morphology

The morphological transition of alewives, *Alosa pseudoharengus* (Wilson), in isolated freshwater ecosystems provides a well-documented empirical illustration of the relationship between functional trait evolution in fish and changes in the zooplankton communities of the fishes’ environment [3]. Over a period of 45–300 years, alewife populations trapped in freshwater ecosystems have undergone a transition from an anadromous to a landlocked morphology, which has been attributed to their interactions with zooplankton communities [11]. Alewives are the dominant force structuring zooplankton communities in eastern North American lakes [12–14]; for example, lakes with landlocked alewives contain smaller-bodied zooplankton than those without alewives [12, 13, 15–18]. Anadromous alewives migrate from salt water to spawn in fresh water and reside in fresh water for approximately six months per year. In contrast, landlocked alewives do not leave freshwater [19]. In lakes with migratory anadromous alewives, the zooplankton community shifts annually between being dominated by large-bodied organisms in the spring and small-bodied organisms in the summer [20]; in lakes with resident landlocked alewives, the zooplankton remain small throughout the year because of stable predation pressure [20].

Alewives have been landlocked in multiple lakes for the past ∼300 years for multiple reasons including dam construction [3, 12, 21]. They were observed in Lake Ontario as early as 1880 [22], and were first recorded in Lake Michigan in 1949 [23]. Today, landlocked alewives are found in the Great Lakes and several of the Finger Lakes of New York [22]. The rapid expansion of alewife populations in the upper Great Lakes disturbed the native fish fauna, causing early summer die-offs of alewives around Milwaukee, Wisconsin, and other cities on the Great Lakes [22]. In Lakes Michigan, Huron, and Ontario, managers are confronted with the challenge of maintaining adequate numbers of alewives to support salmonine stocks while reducing alewife abundance sufficiently to permit the restoration of native fish species [24].

Landlocked alewives show a consistent pattern of life-history divergence from anadromous alewives: landlocked adult alewives have smaller bodies, narrower gapes, smaller inter-gill-raker spacings, lower fecundity, and reach maturity at an earlier age than anadromous alewives [3, 18, 25]. Landlocked alewives also consume exclusively pelagic prey and retain their pelagic niche throughout their lives [26] whereas anadromous alewives consume approximately equal proportions of littoral and pelagic prey, and make an ontogenetic transition from the one to the other [26]. These differences in prey preferences across developmental stages are one potential cause of the morphological differentiation between anadromous and landlocked alewives [26, 27]. The differences in the environmental requirements of landlocked and anadromous populations are currently unknown [28].

Anadromous alewives range from North Carolina to Newfoundland [29]. They spawn along the Atlantic coast from late March through July, and spawning occurs at progressively later dates in more northerly regions [28, 30]. During spawning, 3-to-4-year-old males are abundant, while females dominate among older fish [28]. Males mature earlier than females but have shorter lifespans [28]. Alewives are iteroparous, and females lay nearly all of their eggs during spawning [28]. The alewife absorption stage lasts for 2-5 days [31]. Juveniles typically rear in freshwater for several months before migrating to the ocean between June and November, and they mature at 3-6 years of age [32, 33]. Alewives exhibit olfactory-sensory-driven homing behavior [34] but are not known to show any fidelity to their natal river [35], and there is significant mixing among alewife populations [36].

Alewives play important roles in freshwater and saltwater ecosystems. They feed primarily on zooplankton; the larvae begin to feed on small cladocerans and copepods immediately after developing a functional mouth [28]. They also forage for fish eggs and larvae, and cause native planktivores to go into decline [37–41]. Large alewives feed on small fish, insects, and crustaceans [14, 42]. In turn, they are prey for several piscivores, including bluefish, *Pomatomus saltatrix*, weakfish, *Cynoscion regalis*, striped bass, *Morone saxatilus*, dusky shark, *Carchahinus obscurus*, spiny dogfish, *Squalus acanthias*, salmon, *Salmo salar*, monkfish, *Lophius gastrophysus*, cod, *Gadus morhua*, pollock, *Pollachius virens*, and silver hake, *Merluccius bilinearis* [43]. For stream food webs, anadromous alewives are a potentially important source of marine-derived nitrogen [44]. Because of their ecological importance and low abundance, alewives were declared a Species of Concern by the National Marine Fisheries Service (NMFS) between 2005 and 2007. Harvest restrictions on alewives were imposed in all coastal states, including Massachusetts, Rhode Island, Connecticut, and North Carolina, from 2012 [33].

### Modeling rapid morphological changes of alewives

To improve our understanding of rapid morphological changes in fish, we developed an individual-based model that simulates rapid changes in the body size and gill raker count of a fish species in a hypothetical, size-structured prey community. We adopted a functional trait approach when developing the model; it explicitly describes the relationships between prey size, fish body size, and fish gill-raker count. Ecological power laws [45] were used to define the relationships between the prey-predator body mass ratio and trophic interactions [46–49], between body mass and abundance [50], and between body mass and metabolic rate [51].

Here we report the use of this model to address two questions: (1) How does the impact of alewife populations on prey feed back to impact alewife size and gill raker number under several alternative scenarios? (2) Will the trajectory of landlocked alewives’ morphological evolution change after 150–300 years in freshwater?

## Methods

### Model overview

We combined an individual-based model and difference equations to describe alewives, their prey, and their interactions. The time step of the simulations was one year, and each simulation was allowed to run for 5000 simulated years. Ten replicates of each individual simulation were performed. Model parameter values were derived mainly from empirical studies of alewives.

At each time step, each individual alewife either survives without developing, survives and develops, or dies. Their body size increases stochastically (Algorithm (SI.1)). As an individual grows, their resource requirements for survival, foraging efficiency for different prey sizes, and sexual maturation status changes. The survival of an individual depends on their metabolic rate and foraging ability, the level of resource competition, and prey abundance. A Beverton-Holt function is used to describe the effect of prey mass and fish metabolic rate on density-dependent fish survival (Eq.(SI.16)). There are trade-offs between fish body size and gill-raker spacing that affect the fishes’ foraging, survival, and reproduction. Alewives breed once a year and show iteroparity. The proportion of newborn female offspring is set to 0.5 because the sex ratio of alewives is approximately 1:1 [28]. Anadromous and landlocked alewife populations have a polygynandrous (promiscuous) mating system; therefore, the two ecotypes do not typically co-exist in the same lake [3]. Thus, in the model, each sexually mature female randomly selects a sexually mature male for mating (Algorithm (SI.2)).

**Fig. 1:**
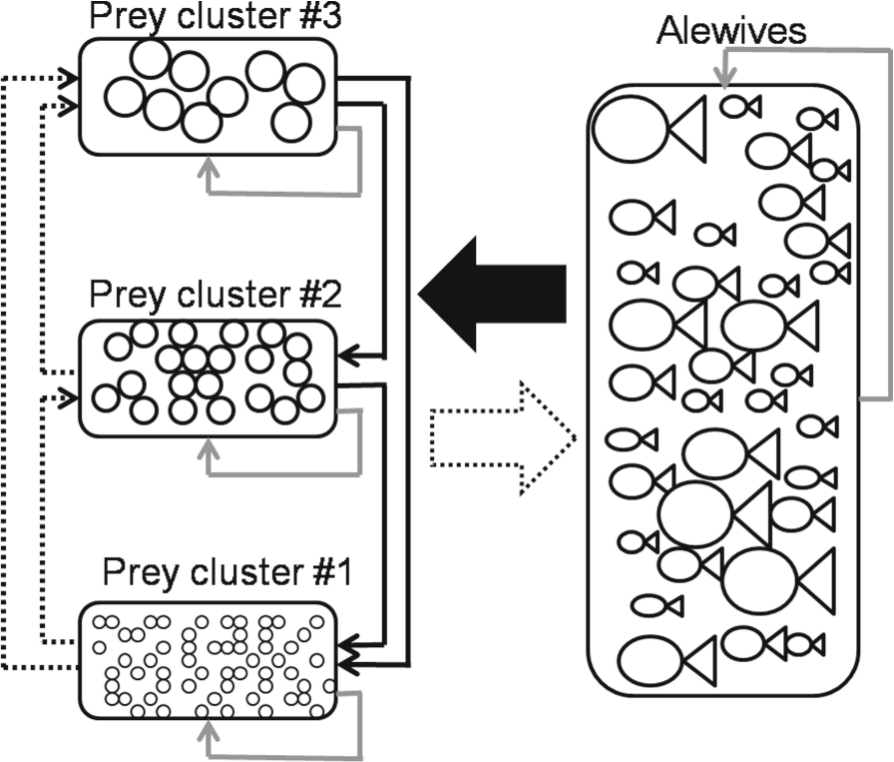
Ecological interactions in the model. A *solid black arrow* from A to B indicates foraging for prey B by predator A. A *dashed black arrow* from A to B indicates an increase in the population growth of predator B foraging for prey A. A *solid grey arrow* indicates competition

The fish forage for different-sized prey; the available prey is conceptualized as a set of size-structured clusters that are described using difference equations. The abundance of prey clusters is influenced by the competition within a prey cluster (i.e. a trophic level), the extent of foraging for smaller-bodied prey clusters, the predation by larger-bodied prey clusters, and foraging by the alewives (Fig. 1).

The following subsections outline the model’s core processes. Additional details of the model are presented in Additional file 1: Supporting information (SI) I, while Additional file 1: Supporting information II provides a list of symbols used in this work and the default input parameter values. We used local sensitivity analyses to determine how uncertainty in the model’s input parameter values affected its outputs. The parameter values used in these local sensitivity analyses are presented in Additional file 1: Supporting information II. The default input parameter values were used at all times in the local sensitivity analyses except where stated otherwise.

### Alewife genetics

Alewives are sexually reproducing diploid organisms. Gill raker number is a heritable trait in most fishes [52, 53]. In our model, the first and second sets of unlinked biallelic quantitative trait loci (QTLs) ({0,1}) influence the body size and the gill-raker count, respectively. A trait is determined by the relative abundance of allele 1 to the total number of alleles in the loci controlling the trait. The default number of loci for a trait is eight, and the mutation rate for alleles is 10^−5^.

### Alewife body size

A stochastic, density-dependent extension of the von Bertalanffy growth function [54] is used to compute the increase in each individual’s body size over each time step. In general, the model assumes that growth is dependent on the growth-affecting QTLs and the environment (Eq.(SI.10)). However, to facilitate analysis of the model’s default behavior, we only simulated the genetic effect on growth. With environmental effects excluded, the parameter *bA* (default value = 0.25) controls the magnitude of each growth-facilitating allele’s effect. We studied the effect of *bA* on alewife morphological evolution by performing a local sensitivity analysis. The default parameter values for the growth process set the average body length of the largest 5-year-old females to approximately the average total body length of adults from three anadromous populations (=260*mm*) and the average body length of the smallest 2-year-old females to a value somewhat lower than the average total body length of adults from three landlocked populations (=97*mm*)[3]. The coefficient of variation for growth, *bcv* (default value = 0.01)(Eq.(SI.8)), controls the individual stochasticity of growth. We tested multiple values (0.001,0.01,0.1,0.2) of *bcv* in a local sensitivity analysis to study the effect of the individual stochasticity of growth on fish morphological evolution.

Some landlocked alewives may spawn at the age of 2 years [55], but 3-to-10-year-old adults dominate anadromous alewife spawning grounds [28, 32, 56, 57]. We therefore assumed that growth-facilitating alleles delayed sexual maturation. Additionally, fertility in many fish species increases with body size [58, 59]. Therefore, in our model, the mean number of offspring produced by a female increases with the female’s body mass (Eq.(SI.30)).

Body size and prey size are positively correlated in many fish species [60–65], including alewives [66]. Accordingly, in our model, an individual’s body size affects the efficiency of their foraging for large prey, *u*. This relationship is described by Eq. (1).

1 $$  u_{i}(t) = 0.01\left[10^{-5.289}\left(bO_{i}(t)^{3.063}\right)\right]  $$

where *bO* is body length (mm), which is used to calculate the available prey mass for an individual (Eq.(SI.15)); 10^−5.289^ and 3.063 are the constant and exponent used to convert body length (mm) to body mass (g), respectively [67]; 0.01 is the default value of the multiplier used to calculate the efficiency of alewives’ foraging for large prey based on body mass. This parameter also regulates the ability of anadromous alewives that are at least one year old to forage for fish eggs (0.1−0.2*g*). Gape size was assumed to be allometrically related to body mass.

### Gill raker

The role of a gill raker apparatus is related to prey retention efficiency [68–70]. Small inter-gill-raker spacings limit the ability of small prey to escape [4], and cross-flow filtering capacity increases with the gill-raker count [4]. For a given number of gill rakers, the inter-gill-raker spacing increases with body size [3, 71, 72]. Moreover, for alewives, the probability of capturing small prey increases as the total body length decreases [71]. Equation (2) describes how the gill-raker spacing, *l* (which controls the efficiency of foraging for small prey), decreases as the gill-raker count increases and/or body size decreases.

2 $$  {} l_{i}(t) = lMn + (lMx - lMn) \left(1-\frac{nml_{i}}{nll \, np} \right) \left[ \frac{w_{i}(t) - wMn}{wMx - wMn} \right]^{eb}  $$

where *nml* is the number of alleles promoting an increased gill-raker count, *nll* is the number of loci controlling gill-raker count, *np* is the number of alleles in a locus, and *eb* determines the effect of body size on gill-raker spacing; the maximum and minimum gill-raker spacings are *lMx* and *lMn*, respectively. The default value for *lMx* is 0.02, which is twice the body mass of large zooplankton (=0.01g). The default value for *lMn* is 0.001, which is 10% of the body mass of large zooplankton. The maximum and minimum fish body masses are *wMx* and *wMn*, respectively. *wMx* and *wMn* were derived from the asymptotic body length of anadromous alewives, *bMx*, and the minimum body length, *bMn* (Eq.(SI.12)), using the alewife body-length-body-mass relationship (body mass (g) =10^−5.289^ [body length (mm) ]^3.063^) [67]. The default value for *bMx* is set to 273.684*mm* in order to make the average body length of the largest 5-year-old females approximately equal to the average total body length of adults from three anadromous populations (=260*mm*) [3].

Low gill-raker spacing values could reduce the efficiency of foraging for large prey because closely-spaced gill rakers are more prone to becoming clogged by large prey than more widely spaced rakers. This reduction in foraging efficiency, *pf*, is modeled by Eq. (3).

3 $$  pf(k,l,lp)= \begin{cases} \left(\frac{l}{k} \right)^{lp} & \text{if}~k>l\\ 1 & \text{if}~k \leq l\\ \end{cases}  $$

where *k* is prey body mass and *lp* (default value= 0.5) controls the decrease in the efficiency of foraging for large prey. Because of the lack of empirical data on *lp*, we performed a local sensitivity analysis to study the effect of *lp* on the evolution of fish morphology. We acknowledge that the reduced efficiency can be attributed to reduced efficiency of the double pump suction mechanism that allows alewives to pull prey into the mouth while pumping the incoming water out through the opercular openings [18].

### Alewives’ foraging

**Fig. 2:**
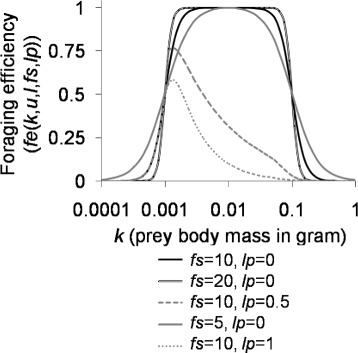
The foraging efficiency (*fe*) function. *u* (=0.1) affected the efficiency of foraging for large prey. *l* (=0.001) affected the efficiency of foraging for small prey. *lp* controlled the effect of gill-raker spacing on foraging for large prey. *fs* controlled the decrease in the efficiency of foraging for very large or very small prey

Equation (4) defines the effects of fish body size and gill-raker spacing on alewives’ foraging for different-sized prey (Fig. 2).

4 $$  \begin{aligned} &{}fe(k,u,l,fs,lp)= \\ &{}\begin{cases} \!pf\!(k,\!l,\!lp)cF(u,\!l,fs)\!\! \left[\! \frac{1}{1+e^{-fs \left(\log_{10}\frac{k}{u} \!\right)}} \right]\! \!\left[\!1\,-\, \frac{1}{1+e^{-fs\left(\log_{10} \frac{k}{l}\!\right)}} \right] &\! \!\!\!\!\text{if}\,u\!>\!l\\ \!0 &\!\! \!\!\mathrm{\!if}\, u \!\leq\! l \end{cases} \end{aligned}  $$

where *fe* is foraging efficiency; *k* is prey body mass; the first and second terms in square brackets describe the decrease in the efficiency of foraging for large and small prey, respectively; *pf* describes the decrease in the efficiency of foraging for large prey due to narrow gill-raker spacing (Eq. (3)); and *fs* describes the decrease in the efficiency of foraging for very large or very small prey. A high *fs* value indicates a rapid decrease in the efficiency of foraging for very large or very small prey. The variables *u* and *l* represent gape size and gill-raker spacing, respectively; a foraging efficiency of 0.5 is achieved when *pf*(*k,l,lp*)=1 and *u*>*l*. When there is no decrease in the efficiency of foraging for large prey due to narrow gill-raker spacing (*pf*(*k,l*,0)=1), *cF* set fish foraging efficiency to the maximum (=1) at $\frac {log_{10} \mathit {u} + log_{10} \mathit {l}}{2}$ (Eq. (5)).

5 $$  \begin{aligned} &{}cF(u,l,fs)=\left[ \frac{2+e^{-fs \left(\tau - \log_{10} l \right)}+e^{-fs \left(\tau -\log_{10} u \right)}}{e^{-fs \left(\tau - \log_{10} l \right)}} \right], \\ &\,\,\,\,\,\quad\tau = \frac{\log_{10} u + \log_{10}l}{2} \end{aligned}  $$

The model describes the type II functional response to alewives’ foraging (Eq.(SI.24)). The effect of the type II functional response on the morphological evolution of fish was studied by performing a sensitivity analysis on *cH*, which determines the handling time. The default *cH* value was 0.1, which reduces the foraging efficiency by 50% when the prey abundance is 10% of that predicted by a body mass-abundance relationship derived from ecological power laws (Eq. (8)) [45, 50].

Traditional foraging theories predict that a prey species providing a better net energy return rate should be chosen more often by a predator species [73–76], which is consistent with the observation that alewives interchangeably switch between filter-feeding and particulate-feeding modes as zooplankton size and density change [77]. In this model, an individual spends more foraging time targeting the prey type with the highest total mass (=abundance × body mass) (Eq.(SI.27)).

### Alewives’ prey (size-structured prey clusters)

The alewives’ prey was divided into multiple size-structured clusters located at different trophic positions. Two or three prey clusters were simulated, with body masses ranging from 10^−3^g to 9.5g. No empirical data were available to determine the number of prey clusters. When more than three prey clusters were simulated using the default parameter settings, one or more prey clusters went extinct. The representative body masses for the three-cluster setting are 0.01g, corresponding to the body mass of large zooplankton; 0.224g, corresponding to the mass of a large fish egg; and 5g, corresponding to the body mass of small organisms foraging for zooplankton and fish eggs. The representative body masses for the two-cluster setting were 0.01g and 0.224g. For simplicity, we assumed that the alewives’ prey was asexually-reproducing diploid organisms whose body mass was determined by an unlinked-biallelic QTL ({0,1}). The default number of loci controlling prey body mass is 10. Therefore, each prey cluster contains 21 discrete body size classes, which are intended to approximate a body size spectrum within each cluster while imposing only a modest increase in computation time. The mutation rate of the allele affecting prey body size is set to 10^−5^. This food web modeling approach differs from conventional food web modeling approaches that assume a continuous size distribution of species because we aimed to study the contemporary evolution of fish in food webs governed by currently unknown mechanisms.

Prey in a cluster was assumed to (1) forage for smaller-bodied clusters (prey), (2) be consumed by larger-bodied clusters (predators) or alewives, and (3) compete with organisms in the same cluster. These trophic interactions and within-trophic competitions are described by the discrete Lotka-Volterra equation (Eq. (6)) [78, 79].

6 $$  rt_{r,rc}(t)= \frac{1+rI_{r,rc}+ pI_{r,rc}(t)}{1+ sI_{r}(t) + nI_{r,rc}(t) + fI_{r,rc}(t) }  $$

where *rt* is the prey abundance before the initiation of dynamic processes within a prey cluster, which is calculated using Eq. (SI.44), and *rI* is the intrinsic growth rate. *rI* is 1 for the prey cluster with the smallest representative body mass, and 0 for other prey clusters. *r* and *rc* are the indices for a prey cluster and a body size class, respectively. *sI*, *nI*, *pI*, and *fI* describe the effect of competition within a cluster, the effect of prey on predators, the effect of predators on prey, and the effect of fish foraging for prey, respectively. To resolve potential problems relating to the increase in the number of model parameters caused by increasing the number of trophic levels, we used allometric relationships derived from ecological power laws (Eq. (7), Eq. (10), Eq. (12)) to set the parameter values for the discrete Lotka-Volterra equation.

### Within-trophic competition among prey

The effect of competition within a prey cluster on prey abundance, *sI*, is given by Eq. (7).

7 $$  sI_{r}(t) = \frac{1}{ra_{r}} \sum\limits_{rc}{rsc_{r,rc}(t-1)}  $$

8 $$  ra_{r}= cA(rMu_{r})^{-eA}  $$

where *ra* is the prey abundance predicted by the body-mass-abundance relationship, *rsc* is the prey abundance after computing the dynamics within the prey cluster using Eq. (SI.44), *cA* (default value = 5.0×10^6^) is a constant for body mass, *rMu* is the representative body mass of a prey cluster, and *eA* is an exponent for body mass. The value of *eA* has been estimated to be 0.84 based on data gathered at Tuesday Lake in 1984 [80], 0.75 based on data gathered at Tuesday Lake in 1986 [80, 81], and 1.1 based on data gathered at the Ythan Estuary [80]. The default value for *eA* was set to 0.75, and the effect of *eA* on alewife morphological evolution was studied by performing a local sensitivity analysis.

### Trophic interactions

For this model, the prey community represents multiple clusters of organisms. Prey organisms forage for organisms in smaller-bodied prey clusters, and they are foraged by organisms in larger-bodied prey clusters. Thus, the prey community for this model resembles a food web, which contains multiple paths of energy flow. The strength of the interaction between prey clusters is treated as a function of the predator-prey body mass ratio [46–49]. Equation (9) defines *nI*, the effect of predation by a larger-bodied prey cluster (predators) on a smaller-bodied prey cluster (prey).

9 $$  {} nI_{r,rc}(t) \,=\,\! \sum\limits_{\substack{rk\\rk \neq r}}{ \!\left[fnc\! \left(\! \left(\sum\limits_{rc}rsc_{rk,rc}(t-1)\right),in_{r,r},\frac{cH_{rk}}{ra_{r}},bf_{r} \right)\right]}  $$

where *fnc* (Eq.(SI.25)) is a functional response and *cH* determines handling time. The default value for *cH* was set at 0.1 to reduce foraging efficiency by 50% when the prey abundance was 10% of that predicted by the body-mass-abundance relationship (Eq. (8)). *bf* is an exponent for prey abundance. Its default value was set to 1 because we assumed a type II functional response in this work. *in* was a scaling factor for the effect of predation by a larger-bodied prey cluster on a smaller-bodied prey cluster. We used Eq. (10) to calculate *in*.

10 $$  in_{r1,r2} = \begin{cases} ci\left(\frac{rMu_{r1}}{rMu_{r2}}\right)^{eI} & \text{if}~rMu_{r2}\geq rMu_{r1}\\ 0 & \text{if}~rMu_{r2}<rMu_{r1}\\ \end{cases}  $$

where *r*1 and *r*2 are indices for a smaller-bodied and a larger-bodied prey cluster, respectively; *ci* (default value = 2.5×10^−6^) is a scaling factor for the predator-prey body mass ratio; and *eI* is an exponent for the predator-prey body mass ratio. The default value for *eI* was 0.25, which is the value obtained by using an allometric scaling relationship to approximate the basal metabolic rate per unit body mass [46, 47, 49].

The effect of the alewives’ foraging for prey was modeled using Eq. (11).

11 $$  fI_{r,rc}(t) = \sum\limits{\left[ fc_{r,s}nc_{r,rc}(t)cn \right]}  $$

12 $$  fc_{r} = cn \; ci\left(\frac{rMu_{r}}{wf}\right)^{eI}  $$

The number of size classes in a cluster, *cn*, is used to scale *fI* such that the *per capita* effect of foraging for a size class is equal to *fc* if an individual spends equal amounts of its foraging time targeting each body size class within the prey cluster that provides the maximum foraging efficiency. The representative body mass for alewives, *wf* (=30.27g), is the mean body mass of female alewives of the largest genotype at the stable-stage distribution; it is calculated from the Leslie matrix shown in Additional file 1: Supporting information I. *nc* is the number of individuals foraging for prey given by Eq.(SI.43).

Equation (13) describes the effect of smaller-bodied prey clusters on larger-bodied prey.

13 $$  pI_{r,rc}(t) = \sum\limits_{\substack{rk\\rk \neq r}}{\left[ip_{rk,r}\sum\limits_{rc}{rsc_{rk,rc}(t-1)} \right]}  $$

14 $$  ip_{r2,r1} = pc \frac{fnc\left(\sum\limits_{rc}rsc_{r1,rc}(t-1),in_{r1,r2},\frac{cH_{r1}}{ra_{r1}},bf_{r1}\right)}{\sum\limits_{rc}rsc_{r1,rc}(t-1)}  $$

where *r*1 and *r*2 are indices for a smaller-bodied prey cluster and a larger-bodied prey cluster, respectively; *pc* (default value = 0.01) was a scaling factor for the predator-prey body mass ratio.

### Initial conditions

During the first 1000 simulated years, only prey were included in the simulation in order to establish stable size-structured prey clusters. Adult alewives (500 males and 500 females) were then introduced into the ecosystem on year 1001. Using the default parameter settings, during the period when only prey was present, the abundance of prey clusters converged to positive numbers. We used the body-mass-abundance relationship (Eq. (8)) to set the prey abundance for the initial time step. The initial abundance distribution of body mass classes in a prey cluster was calculated using the normal distribution (Eq.(SI.46)). The alleles for alewives in the initial population were randomly selected based on known allele frequencies. The default value for the initial frequency of the growth-improving allele (*P*(*A*)_*t*=0_) was 0.99, while that for the frequency of the gill-raker count-increasing allele (*P*(*B*)_*t*=0_) was 0.01. These default parameter settings correspond to a scenario in which a population of anadromous alewives with genetic variation typical of existing anadromous alewives suddenly becomes landlocked. We performed a local sensitivity analysis to study the effect of the initial allele frequencies on the morphological evolution of the fish.

## Results

### Alewife morphological changes before and after the first 250 years

**Fig. 3:**
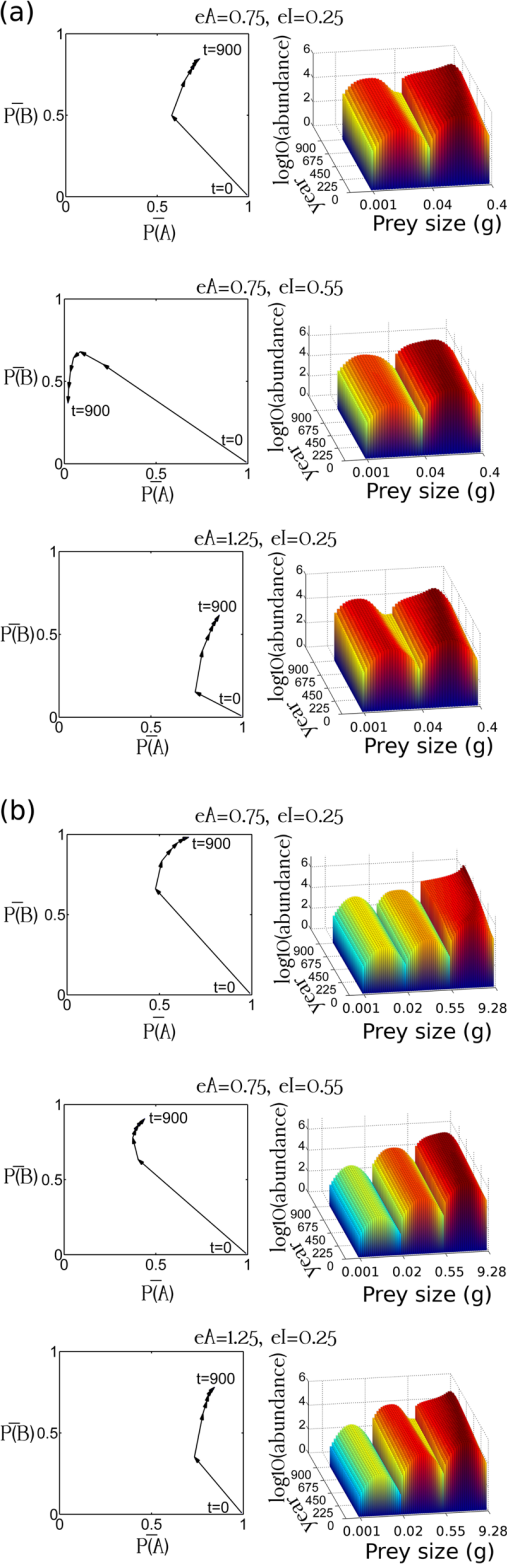
The effect of within-trophic competition and trophic interactions on alewife morphological evolution and prey abundance in ecosystems with two (**a**) or three (**b**) prey clusters. A low *eA* value indicates a low intensity of within-trophic competition. A low *eI* value denotes strong trophic interactions. $\bar {P(A)}$ was the mean frequency of the allele improving growth. $\bar {P(B)}$ was the mean frequency of the allele increasing alewives’ gill-raker count. Reductions in alewives’ body size substantially improved the efficiency of their foraging for small prey (*eb*=0.125). Narrow gill-raker spacings did not reduce the efficiency of the alewives’ foraging for large prey (*lp*=0). Allele frequencies were recorded every 150 years in this figure

For the first 150–250 years after the alewives were introduced into the ecosystems, their growth (*P*(*A*)) decreased monotonically and their gill-raker counts (*P*(*B*)) increased monotonically under most combinations of trophic structure configuration (2 or 3 prey clusters), within-trophic competition level (*eA*), and trophic interactions (*eI*). After the first 150–250 years, however, the alewives exhibited divergent evolutionary trajectories under different trophic settings, and some evolutionary trajectories reversed (Fig. 3). In general, after the first 1000 years, the directions of evolutionary changes in the body size and gill raker count were similar to those during years 250–1000 after introduction. Nevertheless, under certain conditions, the directions of evolutionary changes after the first ∼1000 years changed noticeably from those during years 250–1000. Such deviations were observed when: (1) within-trophic competition was weak (*eA*=0.75), (2) trophic interactions were strong (*eI*=0.25), (3) reductions in body size substantially increased the alewives’ success at foraging for small prey (*eb*=0.125), and (4) narrow gill-raker spacings had moderate negative effects on the alewives’ foraging for large prey (*lp*=0.5).

Under these conditions, the evolutionary trajectory for one morphological trait reversed during the first 150–250 years, and that for the other morphological trait reversed by approximately year 1000. These results show that knowledge of the food web’s structure and the relationships between organisms at different trophic levels is essential for predicting the body size and gill-raker count of landlocked alewives 250 years after their introduction.

**Fig. 4:**
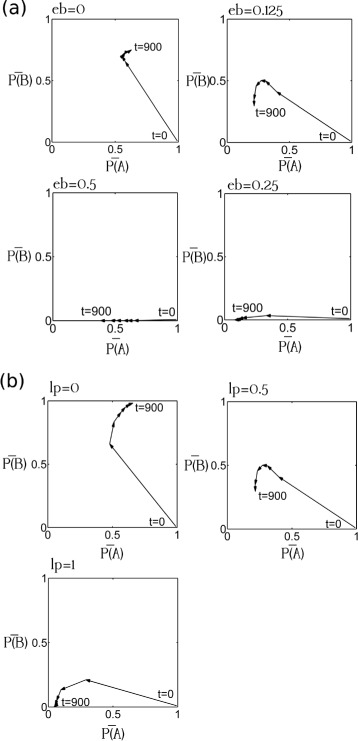
Alewife morphological evolution affected by **a** the effect of body size on foraging for small prey and **b** the effect of clogged gill rakers on foraging for large prey. A low *eb* value indicated that alewives’ small body size substantially improved the efficiency of their foraging for small prey. A high *lp* value indicated that alewives’ small gill-raker spacing greatly undermine the efficiency of their foraging for large prey. The intensity of within-trophic competitions was low (*eA*=0.75). *μ*_*P*(*A*)_ was the mean frequency of the allele improving alewives’ growth. $\bar {P(B)}$ was the mean frequency of the allele increasing alewives’ gill-raker number. Trophic interaction were strong (*eI*=0.25). Alewives’ small gill-raker spacing moderately undermined the efficiency of their foraging for large prey (*lp*=0.5). Allele frequencies were recorded every 150 years in this figure

Over the first 250 years, there were three clear ways in which changes in the alewives’ body size, gill raker spacing, and gill raker count affected their morphological evolutionary trajectory. First, in cases where reductions in the alewives’ body size substantially improved their foraging for small prey (*eb*≤0.25), their gill-raker count increased ((*P*(*B*)_*t*=300_−*P*(*B*)_*t*=0_)>0.1) (Fig. 4). Second, as the effect size of the allele controlling the alewives’ growth decreased (i.e. as *bA* increased), their gill-raker count also increased except in cases where a very small decrease in body size substantially improved their foraging for small prey (*eb*=0). Third, the rate of change of the gill-raker count increased as small gill-raker spacings became less of a barrier to successful foraging for large prey. For example, there was a monotonic decrease in mean growth and a monotonic increase in the mean gill-raker count over the first 150–250 years when (1) reductions in alewife body size substantially improved their foraging for small prey (*eb*≤0.25) and (2) narrow gill-raker spacings had no or only moderate negative effects on foraging for large prey (*lp*=0 or 0.5). Nevertheless, these trends in the evolution of body size and gill-raker count were not observed in cases where (1) there were three prey clusters, (2) trophic interactions were strong (*eI*=0.25), (3) within-trophic competition was weak (*eA*=0.75), and (4) the efficiency of foraging for small prey was substantially increased by very small reductions in alewife body size (*eb*=0).

### Alewives’ foraging efficiency for very large or very small prey

The gill-raker number increased only in cases where the decrease in the alewives’ foraging efficiency for very large or very small prey was moderate (*fs*=10) or fast (*fs*=20). For the first 150 years, the decreases in the alewives’ growth and the increases in their gill-raker numbers were greater when *fs*=20 than when *fs*=10 (Additional file 1: Figure SI.3.1).

### The functional response

Changing the handling time in the type II functional response (*ht*=0,0.05,0.1,0.25) did not appreciably affect the evolutionary trajectory under most of the studied trophic settings. However, the handling time did affect the evolution of the alewives’ body size and gill-raker number when (1) there were three prey clusters, (2) within-trophic competition was weak (*eA*=0.75), (3) trophic interactions were strong (*eI*=0.25), (4) reductions in the alewives’ body size led to substantial improvements in their foraging for small prey (*eb*=0.125), and (5) narrow gill-raker spacings had a moderate negative effect on the alewives’ foraging for large prey (*lp*=0.5) (Additional file 1: Figure SI.3.2).

### Stochasticity in fish morphological evolution

**Fig. 5:**
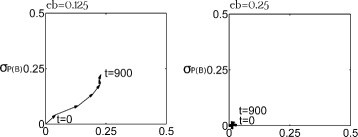
Stochasticity in alewife morphological evolution. A low *eb* value indicated that alewives’ small body size substantially improved the efficiency of their foraging for small prey. *θ*_*P*(*A*)_ was the standard deviation of the frequency of the allele improving alewives’ growth. *θ*_*P*(*B*)_ was the standard deviation of the frequency of the allele increasing alewives’ gill-raker number. The intensity of within-trophic competitions was moderate (*eA*=1). Trophic interaction were moderate (*eI*=0.4). Alewives’ small gill-raker spacing did not undermine the efficiency of their foraging for large prey (*lp*=0). There were three prey clusters. Allele frequencies were recorded every 150 years in this figure

The standard deviations of allele frequencies were generally small (*σ*_*P*(*A*)_<0.05 & *σ*_*P*(*B*)_<0.05) for the first 300 years when reductions in the alewives’ body size moderately improved their foraging for small prey (*eb*>=0.5) or when very small reductions in their body size substantially improved their foraging for small prey (*eb*=0). Nonetheless, in other cases the standard deviations of allele frequencies over the first 300 years were above 0.1. They continued increasing after the first 300 years under several trophic settings when body size reductions substantially improved the alewives’ foraging for small prey (*eb*=0.125 or 0.25) and narrow gill-raker spacings had no effect or only moderate negative effects on their foraging for large prey (*lp*=0 or 0.5) (Fig. 5).

### Individual stochasticity of growth

Different degrees of individual stochasticity of alewife growth produced similar morphological evolutionary trajectories when there were two prey clusters. In contrast, different degrees of individual growth stochasticity resulted in multiple evolutionary morphological trajectories after the first 150–250 years under most trophic settings featuring three prey clusters. Evolutionary trajectories simulated under high individual growth stochasticity (*bcv*=0.2) differed from those under intermediate-or-low individual growth stochasticity (*bcv*=0.001,0.01,0.1) when there were weak or moderate levels of within-trophic competition (*eA*=0.75 or 1) and strong trophic interactions (*eI*=0.25) (Additional file 1: Figure SI.3.3). In addition, evolutionary trajectories simulated under high or low individual growth stochasticity (*bcv*=0.2 or 0.001) differed from those simulated under intermediate individual growth stochasticity (*bcv*=0.01,0.1) when there was strong within-trophic competition (*eA*=1.25) and weak trophic interactions (*eI*=0.55).

### Initial morphology of alewives

**Fig. 6:**
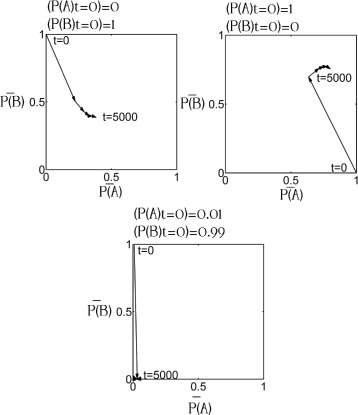
The effect of the initial allele frequencies on alewife morphological evolution. *P*(*A*)_*t*=0_ was the initial frequency of the allele improving alewives’ growth. *P*(*B*)_*t*=0_ was the initial frequency of the allele increasing alewives’ gill-raker number. $\bar {P(A)}$ was the mean frequency of the allele improving alewives’ growth. $\bar {P(B)}$ was the mean frequency of the allele increasing alewives’ gill-raker number. The intensity of within-trophic competitions was low (*eA*=0.75). Trophic interaction were strong (*eI*=0.25). The reduction in alewives’ body size substantially improved the efficiency of their foraging for small prey (*eb*=0.125). Alewives’ small gill-raker spacing moderately undermined the efficiency of their foraging for large prey (*lp*=0.5). Allele frequencies were recorded every 1000 years in this figure

For the first 150–250 years, the alewives’ body size decreased and their gill-raker number increased when a majority of the initially landlocked alewives had large bodies (*P*(*A*)_*t*=0_≥0.99) and a low gill-raker counts (*P*(*B*)_*t*=0_≤0.01), regardless of the initial level of genetic variation. After the first 150–250 years, the evolutionary trajectories simulated under a high initial genetic variation ((*P*(*A*)_*t*=0_=0.99,*P*(*B*)_*t*=0_=0.01) differed from those simulated under low initial genetic variation (*P*(*A*)_*t*=0_=1,*P*(*B*)_*t*=0_=0; *P*(*A*)_*t*=0_=0.9999,*P*(*B*)_*t*=0_=0.0001; *P*(*A*)_*t*=0_=0.999,*P*(*B*)_*t*=0_=0.001) (Fig. 6). Under most trophic settings, the two different initial allele frequency combinations (large-body-and-low-gill-raker-number, i.e. *P*(*A*)_*t*=0_≥0.99, *P*(*B*)_*t*=0_≤0.01, and small-body-and-high-gill-raker-count, i.e. *P*(*A*)_*t*=0_≥0.01, *P*(*B*)_*t*=0_≤0.99) resulted in different allele frequencies within 5000 years. The two sets of initial allele frequency settings, however, resulted in similar allele frequencies (*P*(*A*)_*t*=0_∼0.8,*P*(*B*)_*t*=0_∼0.4) after 5000 years when there were three prey clusters, strong within-trophic competition (*eA*=1.25), and strong trophic interactions (*eI*=0.25).

## Discussion

### Changes in low trophic levels

A reasonable description of multiple, low-trophic-level interactions is indispensable for inferring the abundance and morphology of prey species, which can strongly affect the evolution of fish. This is particularly important when considering small and isolated environments because fish can induce rapid low-trophic-level changes in such contexts. For example, in addition to the well-established changes in zooplankton communities caused by the introduction of alewives, low-trophic-level changes caused by grass carp, *Ctenopharyngodon idella*, silver carp, *Hypophthalmichthys molitrix*, and bighead carp, *Hypophthalmichthys nobilis*, at Lake Donghu in China have been extensively documented [82]. Grass carp were heavily stocked in the lake during the late 1960s and 1970s, and silver carp and bighead carp have been stocked since the 1970s [83]. The stocked fish reduced the density of *Daphnia* from 26.1–35.0 ind./L in 1971–1986 to 0.5–1.3 ind./L in 1987–1996. Moreover, the annual average body length of important prey of silver and bighead carp decreased over this period - from 1.22 mm (1980) to 0.65*mm*(1988) in the case of *Daphnia hyalina*, from 1.33*mm*(1980) to 0.78*mm* (1987) for *Daphnia carinata*, and from 0.81*mm* to 0.61*mm* (1992) for *Daphnia brachyurum* [84–88]. A decrease in the densities of *Daphnia*, rotifers, and protozoans relaxed the competition among small zooplankton, leading to an increase in protozoan biomass [82]. The total annual average densities of zooplankton observed in the 1990s were between 15 and 20 times greater than those recorded in the 1960s, mainly because of this increase in protozoan biomass. The protozoan biomass increase contributed to an increase in the mean annual fish standing crop, from 95.3 kg/ha in the 1960s to 945.1 kg/ha in the 1990s. In this study, alewives were found to affect the abundance and morphology of their prey, which in turn changed the abundance and morphology of the alewives. Complex low-trophic-level interactions can thus alter the abundance and characteristics of fish species. Consequently, it is not sufficient for fisheries management models to merely describe a few predator-prey interactions; a much more detailed description of trophic interactions is required. More accurate and reliable predictions of low-trophic-level changes will improve not only the prediction of fish morphology and abundance but also the management of aquatic ecosystems.

### Ontogenetic changes, trophic interactions, and competition

Selection forces can act differently on fish at different life stages (or ages), and they can change over time. For example, individuals at different life stages (or ages) may forage for different-sized prey, and the strength of competition may differ between life stages. For this reason, we explicitly modeled the ontogenetic changes in the alewives’ foraging characteristics. Describing ontogenetic changes complicated the model but improved the description of resource competition among fish and the representation of trophic interactions.

### Trade-offs between morphological traits

Instead of assuming abstract, theoretical fitness trade-offs, the model explicitly describes trade-offs between body size and gill raker numbers by reflecting findings from empirical studies and potential relationships derived from the mechanical properties of foraging traits. Although this modeling approach increased the number of model parameters, it facilitated the identification of knowledge gaps and model parameterization. In the final model, the body size effect on the gill-raker function (*eb*) and the small gill raker effect on alewives’ foraging for large-bodied prey (*lp*) determine whether the gill-raker number increases during the first 150–250 years. These two properties of alewives’ morphology are trade-offs related to their foraging for different prey types, which can cause divergent natural selection between and within populations exploiting different prey [89–92]. Although both parameters are important for predicting the morphological evolution of alewives, they have not been empirically estimated. Detailed empirical studies on fish foraging will be needed to provide robust parameter estimates and better explanations of the ecological processes related to foraging in fish; once obtained, this information could be used to further improve the prediction of morphological evolution in fish.

### Limitations of this model

This study focused on the effects of species at low trophic levels (prey of alewives) on alewife morphological evolution, and the model does not account for all of the factors that could potentially affect the evolution of alewife body size and gill raker numbers. In particular, to limit complexity, it omits any description of alewives’ predators, even though alewives are common prey for several fish species [35, 93]. In addition, environmental effects on alewives’ growth and sexual maturation were excluded, although they could be accounted for by adjusting the values of relevant parameters such as *ce*. Further studies using this model could be conducted to explore the evolution of fish morphology under different environmental conditions and in the presence of different environmental effects on fish growth and sexual maturation.

## Conclusions

Simulations using a newly developed model showed that alewives’ gill-raker counts only increased during the first 150–250 years after their introduction into a lake if reductions in their body size substantially improved their foraging for small prey. In addition, their gill-raker counts increased to a greater degree as narrow gill-raker spacings became less of a barrier to foraging for large prey. Under most trophic settings, the alewives’ body sizes decreased monotonically over the first 150–250 years, while their gill-raker counts increased monotonically. After the first 150–250 years, however, the alewives exhibited multiple evolutionary trajectories under different trophic settings; in some cases, the evolutionary trajectories established over the first 150–250 years were subsequently reversed. This study suggests that the current morphology of recently (∼300 years)-landlocked alewives may not represent an evolutionarily stable state.

## Additional file

Additional file 1 Contains supporting information I, II, and III. (PDF 833 kb)

## Abbreviations

bA: Parameter controlling the minimum asymptotic body length
bcv: Coefficient variation of body length growth
bf: Exponent for prey density in a functional response
bMn: Minimum body length
bMx: Maximum body length
bO: Body length
cA: Scaling factor for species body mass-abundance relationship
cF: The function controlling the foraging efficiency at the midpoint between *log*_10_ *tu* and *log*_10_ *tl* to 1 when *lp*=0
cH: Parameter controlling handling time
ci: scaling factor for *in*
cn: Number of size classes for a prey cluster
eA: Exponent for body-mass-abundance relation
eb: Effect of body size on fish’s foraging for small prey
eI: Exponent for predator-prey body mass ratio
fc: A scaling factor for the effect of foraging by an individual fish on a size class of a prey cluster
fe: Fish foraging efficiency
fI: Effect of fish foraging for prey
fnc: Functional response
fs: Parameter controlling the difference between the efficiency of foraging on small (or the large) resources and that on medium-sized resources
ht: Handling time
in: Coefficient for the effect of predators on prey
k: Prey body mass
l: Gill raker spacing
lMn: Minimum value for *l*
lMx: Maximum value for *l*
lp: Parameter controlling a decrease in fish foraging efficiency due to small gill-raker spacing
nc: Number of individuals foraging for a resource
nI: Effect of prey on predators
nll: Number of loci controlling the lower-foraging bound
nml: Number of alleles lowering the lower-foraging bound
np: Number of alleles for a locus
NMFS: National Marine Fisheries Service
*P*(*A*): Frequency of the allele increasing body size
*P*(*B*): Frequency of the allele decreasing gill-raker spacing
pc: Scaling factor for ip
pf: Decrease in the foraging efficacy caused by small gill raker spacing
pI: Effect of predators on prey
QTL: Quantitative trait locus
r: Prey cluster
ra: Expected abundance of a prey cluster
rc: A size class in a prey cluster
rI: Intrinsic growth rate
rMu: Representative body mass of a prey cluster
rsc: Abundance of prey in a size class of a prey cluster after the genetic mutation of prey
rt: Abundance of a resource before mutation
*r*1: A prey cluster
*r*2: A prey cluster
sI: Effect of competition within a cluster
SI: Supporting information
u: Gape size
wf: Representative body mass
wMx: Maximum fish body masses
wMn: Minimum fish body masses
*σ*_*P*_: Standard deviations of allele frequency
