# Supplementary material for: Eco-evolution in size-structured ecosystems: simulation case study of rapid morphological changes in alewife
Source: BMC Evol Biol. 2017 Feb 27;17:58. doi: 10.1186/s12862-017-0912-4 (PMC5327535; doi:10.1186/s12862-017-0912-4)

Eco-Evolution in Size-Structured Ecosystems: Simulation Case Study of Rapid Morphological Changes in Alewife

Jung koo Kang & Xavier Thibert-Plante

Supporting information I: Model description

1. Overview

We developed a simulation model to study fish morphological evolution in size-structured resources (prey). We combined an individual-based model and difference equations to describe alewives, prey of the alewives, and their ecological interactions. The descriptions of fish survival, development, foraging, and reproduction are shown in the second section. Prey of alewives was modeled as multiple, different-sized clusters, and difference equations described it. Prey abundance was affected by the competition within a prey cluster, the foraging for smaller-bodied prey clusters, the predation by larger-bodied prey clusters, and the foraging by alewives. The descriptions of the

Figure SI.1.1: Flow diagram of a model for studying alewife morphological evolution. The equation numbers are in []. The algorithm numbers are in ().

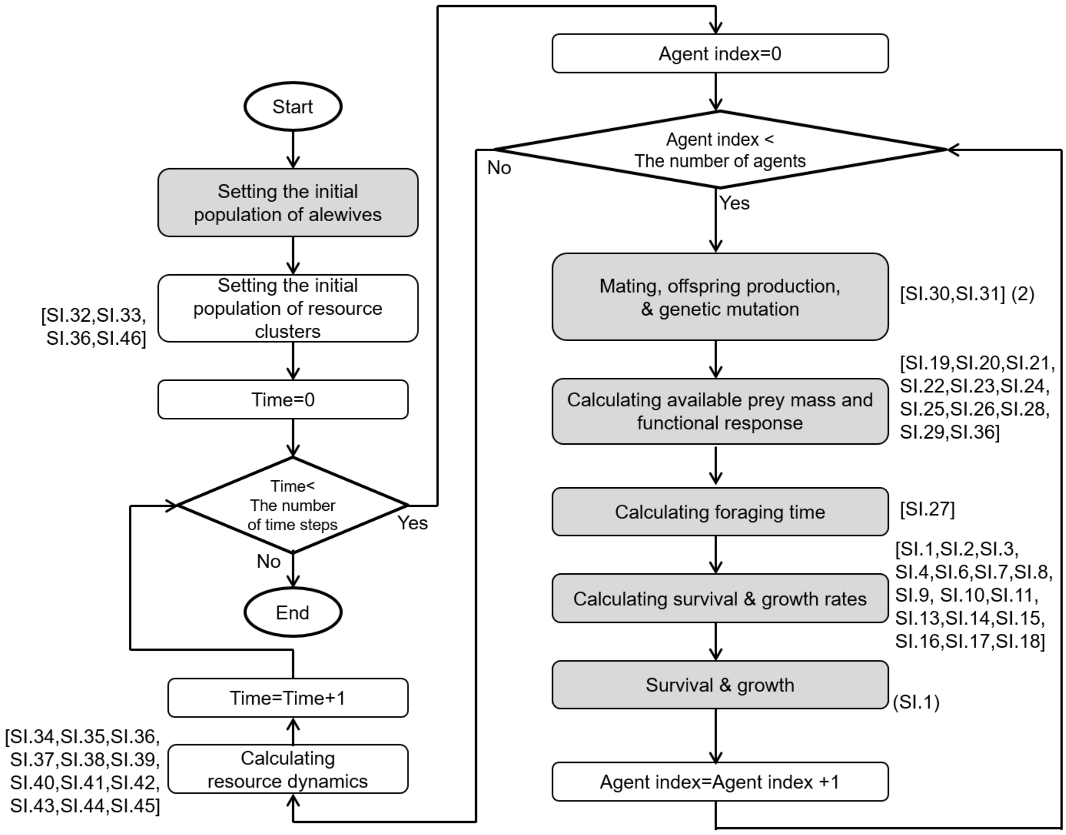

structure and dynamics of prey clusters are shown in the third section. The last section shows the initial conditions for simulations. Fig.SI.1.1 shows the processes in this model, and model symbols are shown in Table SI.2.1 in supporting information II.

2. Fish vital processes

An individual 1) survived at the current life stage, 2) survived and developed to the next life stage, or 3) died for each time step. We implemented Algorithm (SI.1) to simulate these vital processes. To facilitate the computation, individuals were sorted into 250 equal-width, body-length bins, which ranged from 0 to the maximum mean asymptotic body length, bMx. The default value for bMx was $273.684mm$ to set the average body length of 5-year-old females of the largest genotype to approximately the average total body length of adults from three anadromous alewife populations ($= 260mm$) (Palkovacs *et al.* , 2008).

Update the age of the individual ($age_i = age_i + time\,step$).
Calculate m_i by using Eq. (SI.14).
Calculate $ddS_i(t)$) by using Eq. (SI.16).
Calculate $sm_i(t)$ by using Eq. (SI.29).
$u \sim$ Uniform distribution $[0, 1]$
if *the individual is not reproductively matured* **then**
  if $u < (P_{x,sg_i} ddS_i(t))$ **then**
    The individual, i, survives at the current life stage.
    $u \sim$ Uniform distribution $[0, 1]$
    if $u < sm_i(t)$ **then**
      $sm_i = true$
    end
  else if $u < [(P_{x,sg_i} + G_{x,sg_i}) ddS_i(t)]$ **then**
    The individual, i, survives and develops to the next life stage by setting $sg_i = sg_i + 1$.
    $u \sim$ Uniform distribution $[0, 1]$
    if $u < sm_i(t)$ **then**
      $sm_i = true$
    end
  else
    The individual, i, dies.
  end
else
  if $u < [(P_{x,sg_i} + G_{x,sg_i}) ddS_i(t)]$ **then**
    The individual, i, survives at the current life stage.
  else
    the individual, i, dies.
  end
end
if *the individual is alive* **then**
  Update the body length, b_i, by using Eq. (SI.1).
end

Algorithm 1: Survival and development process for an individual. P was the probability of an individual surviving at the current life stage for a time step without density-dependent effects; G was the probability of an individual surviving and developing to the next life stage for a time step without density-dependent effects; sg was the index for a life stage; x was the index for a sex; i was the index for an individual; m was the metabolic rate; ddS was the density-dependent survival rate; sm was the probability that an individual sexually matured; sm was the status of sexual maturation.

We used density-dependent and density-independent rates for fish vital processes. The default values for the density-independent survival and growth rates of female alewives are shown in a form of a stage-classified matrix population model. The matrix does not contain fertility, which is described in the reproduction subsection, because fertility was not fixed.

$$\begin{bmatrix} P_{1,1}(=0) & 0 & 0 & 0 & 0 \\ G_{1,1}(=0.670) & P_{1,2}(=0) & 0 & 0 & 0 \\ 0 & G_{1,2}(=0.670) & P_{1,3}(=0) & 0 & 0 \\ 0 & 0 & G_{1,3}(=0.670) & P_{1,4}(=0) & 0 \\ 0 & 0 & 0 & G_{1,4}(=0.670) & P_{1,5}(=0.6281) \end{bmatrix}$$

The growth rates, G, for the first to the fifth life stages were derived from instantaneous juvenile natural mortality estimated by Gibson et al. (Gibson & Myers, 2004). The survival rate, P, for the fifth life stage was derived from median instantaneous adult natural mortality estimated by Gibson et al. (Gibson & Myers, 2003).

Male alewives sexually mature earlier than the females (Mullen *et al.* , 1986). For this reason, females had four immature life stages, and males had three immature life stages. The matrix below shows vital rates for male

alewives.

$$\begin{bmatrix} P_{1,1}(=0) & 0 & 0 & 0 \\ G_{1,1}(=0.670) & P_{1,2}(=0) & 0 & 0 \\ 0 & G_{1,2}(=0.670) & P_{1,3}(=0) & 0 \\ 0 & 0 & G_{1,3}(=0.670) & P_{1,4}(=0.6281) \end{bmatrix}$$

2.1. Body growth

The body length of an individual increased for each time step by using Eq. (SI.1).

$$b(t)_i = b(t-1) + \Delta b(t)_i \tag{SI.1}$$

where, b was total body length (mm). Body length at age 0 was 0. Body growth, $\Delta b(t)_i$, was calculated by using Eq. (SI.2).

$$\Delta b(t)_i = bB_i(t) + bD_i(t) \tag{SI.2}$$

where, bB was the baseline growth; bD was the growth affected by genetics, environments, and individual variation. We used the von Bertalanffy growth function to calculate bB and bD. The baseline growth was given by Eq. (SI.3).

$$bB_i(t) = bMx(1 - bA)kV_i(t) \tag{SI.3}$$

where, bMx was the maximum mean asymptotic body length; bA affected the minimum asymptotic body length ($= bMx_s(1 - bA)$). When there was

no environmental effect on growth ($ce = 0$) (Eq. (SI.10)), bA determined only the effect size of the allele improving growth. The default value for bA was 0.25 to make the body length of the smallest genotype at age 2 shorter than the average total body length of adults from three landlocked alewife populations ($= 97mm$)(Palkovacs *et al.* , 2008). kV was the degree of growth calculated from the von Bertalanffy growth function (Eq. (SI.4)).

$$kV_i(t) = e^{ev\frac{age_i(t-1)}{tx_x}} - e^{ev\frac{age_i(t)}{tx_x}} \tag{SI.4}$$

where, age was the age of an individual; x was the index for sex; tx was the age (year) of the largest genotype becoming sexually matured. During spawning, 3-to-4-year-old males are abundant, and females dominate among older fish (Mullen *et al.* , 1986). To reflect this, the default value for tx was 5 for females and 4 for males. The default value for an exponent, ev, was $log_e 0.05$ to complete 95 % of growth at tx when ed, which was the environmental effect on growth (Eq. (SI.13)), was constant over time.

Based on the physiological concepts underlying the von Bertalanffy growth function, density-dependent growth mediated by resource competition is expected to affect the asymptotic size of fish, but not the growth rate at which this size is approached (Beverton & Holt, 1957; von Bertalanffy, 1960), and this was supported by theoretical and empirical studies (Walters & Post, 1993; Lorenzen, 1996; Shin & Rochet, 1998); for example, Lorenzen (Lorenzen, 1996, 2000) showed that a simple function with the asymptotic length de-

fined as a linear function of population biomass density described the density-dependent growth of fishes in various pond experiments ($L_{\infty B} = L_{\infty L} - gB$, where, $L_{\infty B}$, $L_{\infty L}$, g, and B were the asymptotic body length, the limiting asymptotic body length, the decline in asymptotic body length per unit of biomass density, and biomass density, respectively). We used a stochastic, density-dependent extension of the von Bertalanffy growth function (Eq. (SI.5)) to describe fish growth in variable resources.

$$bD_i(t) \sim ln\mathcal{N}(l_i, v_i) \tag{SI.5}$$

where, $bD_i(t)$, growth, was a random deviate drawn from the log-normal distribution ($ln\mathcal{N}$) with the location, l_i, and the scale, v_i. To prevent biologically implausible growth, only uniform random deviates between 3.15×10^{-5} and $1 - 3.15 \times 10^{-5}$, which were the quantiles within four standard deviations of the normal distribution, were used to draw random deviates from the log-normal distribution. l and v were calculated by using the mean, μ, and the standard deviation, σ, of growth for a time step.

$$l_i = Log_e(\mu(t)_i) - \frac{1}{2}(v_i)^2 \tag{SI.6}$$

$$v_i = Log_e\left(1 + \frac{\sigma^2}{\mu(t)_i}\right) \tag{SI.7}$$

Standard deviation of growth, σ, was the product of mean growth, μ, and

the coefficient variation of growth, bcv.

$$\sigma = \mu(t)_i bcv \tag{SI.8}$$

bcv (default value = 0.01)determined the individual variation in growth. We used a local sensitivity analysis to study the effect of the individual variation in growth on fish morphological evolution. Mean growth, $\mu(t)_i$, was calculated by using Eq. (SI.9).

$$\mu(t)_i = bMx\ cV_i(t)kV_i(t) \tag{SI.9}$$

where, bMx was the maximum mean asymptotic body length; kV was the degree of growth calculated from the von Bertalanffy growth function. The asymptotic body length, cV reflected the genetic and environmental effects on growth, and it was modeled as Eq. (SI.10).

$$cV_i(t) = bA\{bR + (1 - bR)[(1 - ce)A_i + ce\ ed_i(t)]\} \tag{SI.10}$$

where, bA controlled the minimum asymptotic body length ($0 \leq bA \leq 1$); bR (default value = 0.1) was the minimum growth; ed was the effect of resource mass on body length growth, and it is described in the following subsection; ce determined the environmental effect on growth and sexual maturation. For this study, ce was 0 (1) to focus on studying the default model behavior without the environmental effect on growth and sexual maturation and (2)

to limit the scope of this study. The genetic effect on growth, A, was given by Eq. (SI.11) .

$$A_i = \frac{nmu_i}{nlu\, np} \tag{SI.11}$$

where, nmu was the number of alleles improving growth; nlu and np were the number of loci affecting growth and the number of alleles per locus, respectively.

The minimum body length, bMn, was given by Eq. (SI.12).

$$bMn = bMx\ kV(1)[1 - (bA(1 - bR))] \tag{SI.12}$$

where, bA determined the minimum body length; bR was the minimum degree of growth; $kV(1)$ was the growth for 1-year-old fish calculated from the von Bertalanffy function.

2.2. Environmental effect

The effect of available prey mass on growth, ed, was calculated by using Eq. (SI.13).

$$ed_i(t) = e^{dg \frac{ir_i(t)}{cD\ m_i(t)}} \tag{SI.13}$$

where, dg was a scaling factor. The default value for dg was $log_e 0.75$ to set ed to 0.75 when no density-dependent mortality occur ($\frac{ir_i(t)}{cD\ m_i(t)} = 1$). Density-dependent mortality is described in the following subsection. ir was available prey mass for an individual.

The metabolic rate of an individual, m, was a function of body mass (Kleiber's law (Kleiber, 1932)), and it was given by Eq. (SI.14).

$$m_i(t) = \left[10^{-5.289}(bO_i(t))^{3.063}\right]^{\frac{3}{4}} \tag{SI.14}$$

where, $10^{-5.289}$ and 3.063 were a scaling factor and an exponent for converting body length (mm) to body mass (g) for alewives, respectively (Schneider, 2000). $\frac{3}{4}$ was the exponent for converting body mass to metabolic rate. The body length (mm) under the optimum resources, bO, was calculated by using Eq. (SI.15).

$$bO_i(t) = b(t-1) + \{(1-bA) + bA[(1-ce)A_i + ce]\}\, kV_i(t) \tag{SI.15}$$

where, b was body length (mm); bA determined the minimum asymptotic body length; ce determined the environmental effect on growth and sexual maturation; A was the genetic effect on growth; kV was the growth based on the von Bertalanffy growth function.

2.3. Density-dependent survival

A large body should be maintained by a sufficient food supply (Rosenzweig, 1968; Olalla-Trraga *et al.* , 2006); accordingly, high metabolic rate of a large body can negatively affect survival if a food supply is limited. We reflected these relationships to describe the density-dependent survival of fishes. Density-dependent survival rate, ddS, was a function of a metabolic

rate, m, and the prey mass accessible to an individual, ir. The Beverton-Holt function was used to calculate ddS (Eq. (SI.16)).

$$ddS_i(t) = \frac{1}{1 + cD\frac{m_i(t)}{ir_i(t)}} \tag{SI.16}$$

where, cD was the scaling factor for density-dependent survival. The default value for cD was 10, which was an arbitrary value because of the lack of empirical data. m was a metabolic rate. The prey mass accessible to an individual, ir, was affected by prey abundance, rsc, prey body mass, rMu, a functional response, frp (Eq. (SI.24)), and their foraging time for prey, ft (Eq. (SI.27)). We used Eq. (SI.17) to calculate ir.

$$ir_i(t) = \sum_r \sum_{rc} \frac{rsc_{r,rc}(t-1) rMu_{r,rc} frp_{i,r,rc}(t) ft_{i,r,rc}(t)}{cp_{r,rc}(t)} \tag{SI.17}$$

where, i was the index for an individual; r was the index for a prey cluster; rc was the index for a size class of a prey cluster; $cp_{r,rc}(t)$ represented the resource competition among alewives.

$$cp_{r,rc}(t) = \sum_i frp_{i,r,rc}(t) ft_{i,r,rc}(t) \tag{SI.18}$$

We acknowledged that other density-dependent-survival functions can be used to describe the relationship between density-dependent survival and prey mass.

2.4. Fish's Foraging

2.4.1. Body size and foraging for large prey. Body size and prey size are positively correlated for many fish species (Keast & Webb, 1966; Popova, 1967, 1978; Nielsen, 1980; Persson, 1990; Juanes *et al.* , 1994). This trend is also found in alewives (Kohler & Ney, 1980). To reflect this, for this model, fish body size limited the efficiency of their foraging for large prey. This was modeled as Eq. (SI.19).

$$u_i(t) = 0.01[10^{-5.289}(bO_i(t)^{3.063})] \tag{SI.19}$$

where, u, a paramteter for the sigmoid function for limiting the efficiency of their foraging for large prey; bO was the body length (mm) used for calculating the available prey mass for an individual (Eq. (SI.15)); $10^{-5.289}$ and 3.063 were a scaling factor and an exponent for converting fish body length (mm) to body mass (g), respectively (Schneider, 2000). The body mass was multiplied by 0.01 to allow one-year-old anadromous alewives to forage for fish eggs $(0.1 - 0.2g)$. Fish gape size was assumed to be allometrically related to the body mass.

2.4.2. Gill-rakers and foraging for small resources. The role of a gill raker apparatus is related to prey retention efficiency (Drenner, 1977; Sanderson *et al.* , 2001; Smith & Sanderson, 2008). An increasing number of gill rakers enhances cross-flow filtering, and closely spaced gill rakers limit

the escape possibility of small prey (Kahilainen *et al.* , 2011); thus, a high number of long gill rakers is common in planktivorous fish species and morphs although benthic species and morphs usually display a low number of shorter gill rakers (Janssen, 1980; Gibson, 1988; Schluter & McPhail, 1992; Langeland & Nst, 1995; Robinson & Parsons, 2002). For this model, gill-raker spacing, which determined the efficiency of their foraging for small prey, l, improved as the number of gill rakers increased (Kahilainen *et al.* , 2011) and/or body size decreased (MacNeill & Brandt, 1990; Friedland *et al.* , 2006; Palkovacs *et al.* , 2008). These relationships were modeled as Eq. (SI.20).

$$l_i(t) = lMn + (lMx - lMn)(1 - B)\left[\frac{bO_i(t) - wMn}{wMx - wMn}\right]^{eb} \tag{SI.20}$$

where, *nml* was the number of the alleles increasing a gill-raker number; B $(= \frac{nml_i}{nll\, np})$ was the genetic effect on a gill-raker number; *nll* was the number of the loci affecting a gill-raker number; *np* was the number of alleles for a locus; *eb* (default value = 0.125) determined the effect of fish body size on their foraging for small prey. Because *eb* has not been empirically estimated, we used a sensitivity analysis to study the effect of *eb* on fish morphological evolution. The maximum and minimum values for gill-raker spacing, l, were *lMx* and *lMn*, respectively. The default value for *lMx* was 0.02, which was twice the body mass of large zooplankton (= 0.01g). The default value for *lMn* was 0.001, which was 10% of large zooplankton body mass. The maximum and minimum body masses were *wMx* and *wMn*, respectively.

wMx and wMn were derived from the asymptotic body length, bMx, and the minimum body length, bMn, by using the alewife body-length-body-mass relationship (body mass (g) = $10^{-5.289}$[body length (mm)]$^{3.063}$) (Schneider, 2000).

2.4.3. Clogged gill rakers. Large particles are more likely to clog small inter-gill-raker spacing than small particles; accordingly, small gill-raker spacing can decrease the efficiency of their foraging for large prey. Eq. (SI.21) described a decrease in their foraging efficiency caused by small gill-raker spacing.

$$pf(k, l, lp) = \begin{cases} \left(\frac{l}{k}\right)^{lp} & \text{if } k > l \\ 1 & \text{if } k \leq tl \end{cases} \tag{SI.21}$$

where, lp determined a decrease in the efficiency of their foraging for large prey. We used a local sensitivity analysis to study the effect of lp on the morphological evolution of fish. The default value for lp was 0.5, which was an arbitrary value chosen because of the lack of empirical data.

2.4.4. Foraging efficiency.

Body size and gill-raker spacing affected fishes' foraging for different-sized prey for this model, and this was modeled as Eq. (SI.22) (Fig. 2).

$$fe(k,u,l,fs,lp) = \begin{cases} pf(k,l,lp)cF(u,l,fs)\left[\frac{1}{1+e^{-fs\left(\log_{10}\frac{k}{u}\right)}}\right]\left[1-\frac{1}{1+e^{-fs\left(\log_{10}\frac{k}{l}\right)}}\right] & \text{if } u > l \\ 0 & \text{if } u \le l \end{cases} \tag{SI.22}$$

where, *fe* was foraging efficiency; k was prey body mass; *pf* was the function describing a decrease in their foraging efficiency caused by small gill-raker spacing (See the clogged gill rakers subsection); *fs* determined a decrease in their foraging efficiency at around u, which represented fish body size, and l, which represented gill-raker spacing. The terms inside the first square bracket described the efficiency of their foraging for small prey. When $u > l$, the foraging efficiency was 0.5 at l. The terms inside the second square bracket described the efficiency of their foraging for large prey. When there is no decrease in the efficiency of foraging for large prey due to narrow gill-raker spacing ($pf(k, l, 0) = 1$), *cF* set fish foraging efficiency to the maximum (=1) at $\frac{log_{10}u+log_{10}l}{2}$ (Eq. (SI.23)).

$$cF(u,l,fs) = \left[\frac{2+e^{-fs\left(\frac{\log_{10}u+\log_{10}l}{2}-\log_{10}l\right)}+e^{-fs\left(\frac{\log_{10}u+\log_{10}l}{2}-\log_{10}u\right)}}{e^{-fs\left(\frac{\log_{10}u+\log_{10}l}{2}-\log_{10}l\right)}}\right] \tag{SI.23}$$

Some large predators can demonstrate fast swimming speed and improved visual acuity (Keast & Webb, 1966; Webb, 1976; Beamish, 1978; Hubbs & Blaxter, 1986; Scharf *et al.* , 2000); large preys may escape from predators better due to improved escape response, reaction distance, and swimming performance (Folkvord & Hunter, 1986; Blaxter & Fuiman, 1990; Scharf *et al.* , 2000). These correlations were not modeled to limit the scope of this study.

2.4.5. Functional response. We assumed that fishes showed the type II functional response, which was given by Eq. (SI.24).

$$frp_{i,r,rc}(t) = fnc(rsc_{r,rc}(t-1), fe(rMu_{r,rc}, u_i, l_i), ht_r, bf_{r,rc}) \tag{SI.24}$$

$$fnc(x, af, ht, bf) = \frac{af\, x^{bf}}{1 + (af\, ht\, x^{bf})} \tag{SI.25}$$

where, *frp* was the efficiency of foraging with a functional response; *fnc* was a functional response; *af* was attack rate; *bf* was the exponent for prey density, whose default value was 1 because we assumed the type II functional response by fishes; *ht* was handling time, and it was given by Eq. (SI.36).

$$ht_r = \frac{cH_r cn}{ra_r} \tag{SI.26}$$

where, *cH* was a parameter for handling time; *cn* was the number of size classes in a prey cluster. *ra* was the prey abundance predicted by a body-

mass-abundance relationship of ecological power laws (Marquet, 2002; Jennings & Mackinson, 2003) (Eq. (SI.36)).

2.4.6. Foraging time. Traditional foraging theories predict that a predator should choose a prey species providing a better net energy return rate (Schoener, 1971; Charnov, 1976; Stephens & Krebs, 1986; Scharf *et al.* , 2000). Similarly, alewives interchangeably use filter-feeding and particulate-feeding modes as zooplankton size and density change (Janssen, 1976); therefore, for this model, alewives spent more time for foraging for prey with larger total mass (=abundance × body mass). We used Eq. (SI.27) to calculate the proportion of time that an individual foraging for different-sized prey, *ft*.

$$ft_{i,r,rc}(t) = \frac{frp_{i,r,rc}(t)rMu_{r,rc}}{\sum_{r}\sum_{rc}[frp_{i,r,rc}(t)rMu_{r,rc}]} \tag{SI.27}$$

where, *rMu* was the *per capita* prey body mass.

2.5. Sexual maturation

Landlocked alewives display smaller adult body size and maturity at an earlier age than anadromous alewives (Palkovacs *et al.* , 2008; Palkovacs & Post, 2008; Schielke *et al.* , 2011). Landlocked alewives may spawn as early as 2 years old (Lackey, 1970) although 3-to-10-year-old adults of anadromous alewives dominate spawning grounds (Joseph & Davis, 1965; Marcy Jr, 1969; ONeill, 1980; Mullen *et al.* , 1986). To reflect this correlation between body

size and sexual maturity, we assumed that the alleles increasing fishes' body size delayed their sexual maturation as a trade-off. Besides this pleiotrophic effect, an increase in resource availability generally accelerates fishes' reproductive ontogeny and growth; therefore, fishes mature earlier at larger sizes when their resource is abundant (Trexler, 1989; Reznick *et al.* , 1990). The genetic and environmental effects on sexual maturation, sB, were modeled as Eq. (SI.28).

$$sB_i(t) = (1 - ce)(1 - A_i) + ce\ ed_i(t) \tag{SI.28}$$

where, ce was the parameter determining the environmental effect on fish growth and sexual maturation; A was the genetic effect on fish growth and sexual maturation; ed was the effect of prey mass on fish growth.

For this model, fishes reproduced once they were sexually matured, and the probability of sexual maturation, sm, was given by Eq. (SI.29).

$$sm_i(t) = \begin{cases} 0 & \text{if } sB_i(t) = 0 \ or \ age_i < (tx_x - eag_x) \\ (sB_i(t))^{\frac{1}{eag}} & \text{if } sB_{s,i}(t) > 0 \ and \ (tx_x - eag_x) \leq age_i < tx_x \\ 1 & \text{if } age_i \geq tx_x \end{cases} \tag{SI.29}$$

where, sg was life stage; i was the index for an individual; x was the index for sex; tx was the oldest age for sexual maturation; eag was the duration (years) that an individual had a chance of sexual maturation -1. When

$eag = 0$, all individuals sexually matured at tx.

2.6. Reproduction

Fertility in many fish species increases with body size (Bagenal, 1967; Gross, 1987). We used Eq. (SI.30) to describe this relationship.

$$F_i(t) = fmx \left(\frac{w_i(t)}{wMx} \right)^{frb} \tag{SI.30}$$

where, F was the mean number of newborn female offspring from a mated female for a time step; *fmx*, w, and *wMx* were the maximum fertility, body mass, and the maximum body mass, respectively; *fmx* for alewives was 121.27 to set the maximum annual reproductive rate for three-year-old females of the largest genotype to 85, which was the median of the maximum annual reproductive rate estimated in eight alewife populations with the two-parameter compensatory spawner-recruit Beverton-Holt model (Myers *et al.* , 1999; Barrowman, 2000; Myers *et al.* , 2001; Gibson & Myers, 2004); *frb* (= 0.893)was an exponent for the body-mass-fertility conversion, which was based on the body-mass-fertility relationship in whitefish and vendace (Sandlund *et al.* , 2013). The body-mass-fertility relationship in alewives has not been estimated.

Alewives breed once a year at ponds, lakes, or slow-flowing portions of stream (Scott & Scott, 1988). Alewives show polygynandrous (promiscuous) mating system and iteroparity. Anadromous and landlocked alewives do not

typically co-exist in the same lake (Palkovacs *et al.* , 2008). The polygynandrous mating, oviposition, and fertilization of alewives were simulated by using Algorithm (SI.2).

if *an individual is a sexually-matured female* **then**
  $nf \sim$ discrete normal distribution $(\mu = F_i(t)cX, \sigma^2 = (Fsd_i(t))^2)$
  if $nf > 0$ **then**
    $j = 1$
    while $j < (nf + 1)$ **do**
      Draw one gamete from the chosen male and one gamete from the female individual, and then set the genotype for offspring.
      $u \sim$ Uniform distribution$[0, 1]$
      if $u < fp$ **then**
        The newborn offspring is female.
      else
        The newborn offspring is male.
      end
      $j = j + 1$
    end
  end
end

Algorithm 2: Algorithm for the polygynandrous mating, oviposition, and fertilization of alewives

where, Fsd was the standard deviation of the number of female offspring from a mated female for a time step. nf was the number of offspring from a mated female for a time step. To prevent biologically implausible fertility of fish, only uniform random deviates between 3.15×10^{-5} and $1 - 3.15 \times 10^{-5}$, which were the quantiles within four standard deviations of the normal distribution, were used to draw random deviates from the normal distribution. Instead of the discrete normal distribution, Poisson distribution can be used to draw the number of newborn offspring if the mean and standard deviation are equal. cX was a factor for converting the number of female offspring to the number of male and female offspring. cX was given by Eq. (SI.31).

$$cX = \left[1.0 + \left(\frac{1.0 - fp}{fp} \right) \right] \tag{SI.31}$$

where, *fp* was the proportion of newborn female offspring. The default value for *fp* was 0.5 because the sex ratio of alewives is approximately 1 : 1 (Mullen *et al.* , 1986). For the gamete formation, one of two alleles was randomly chosen for each locus. Genetic mutation in alewives was implemented by switching alleles (0 or 1) with the genetic mutation rate (= 10^{-5}).

3. Size-structured prey clusters (prey of alewives)

We modeled the prey of alewives as multiple, discrete, size-structured clusters with different trophic positions. Instead of using conventional approaches with the assumption of the continuous body size distribution of species, we

took this food web modeling approach to study fish morphological evolution in food webs governed by mechanisms previously not investigated. Prey clusters were sorted by their representative body masses in ascending order. The representative body masses except the maximum (= rMu_{rn}) or minimum (= rMu_1) representative body masses was determined by Eq. (SI.32).

$$rMu_r = \left(\frac{rMu_{rn}}{rMu_1}\right)^{\frac{r-1}{rn-1}} \tag{SI.32}$$

where, r was the index for a prey cluster; rn was the number of prey clusters. For this study, two or three prey clusters were simulated because one or more clusters went extinct when over three clusters were simulated under the default parameter setting. The default value for rMu_{rn} was 5 when there were three prey clusters, and it was 0.224 when there were two prey clusters. The default for rMu_1 was 0.01. Under these settings, the representative body masses for three prey clusters were 0.01g, which represented the body mass of large zooplankton, 0.224g, which represented the body mass of a large fish egg, and 5g, which represented the body mass of small organisms foraging for zooplanktons and fish eggs. Only the smallest two prey clusters were simulated when there were two prey clusters.

The range of body mass of a prey cluster ($[rMu_r - 3wSD_r, rMu_r + 3wSD_r]$) was defined by using the representative body mass, rMu, and the standard deviation of body mass in a prey cluster, wSD. We used Eq. (SI.33) to

calculate wSD.

$$wSD_r = rCV\ rMu_r \tag{SI.33}$$

where, rCV (default value = 0.3)was the coefficient variation of the body mass.

The range of body mass in a prey cluster was divided into equal-width body size classes. For simplicity, we assumed that organisms in prey clusters were diploid and reproduced asexually. In addition, we assumed that unlinked-biallelic QTL determined the body mass of organisms in prey clusters. The default number of the loci affecting prey body mass was 10; therefore, the number of size classes in a prey cluster, cn, was 21 (=the number of alleles at loci $\times$ the number of alleles for a locus +1), which described the prey size spectrum with the moderate increase in computation time.

3.1. Prey dynamics

Trophic interactions and within-trophic competitions affected prey abundance. Prey abundance was negatively influenced by (1) the competition within a prey cluster, (2) the predation by larger-bodied prey clusters, and (3) the predation by alewives; prey abundance was positively influenced by foraging for smaller-bodied prey clusters. We used the discrete Lotka-Volterra equation (Eq. (SI.34)) to describe prey dynamics (Leslie, 1958; Liu & Elaydi, 2001).

$$rt_{r,rc}(t) = \frac{1 + ri_{r,rc} + pI_{r,rc}(t)}{1 + sI_r(t) + nI_{r,rc}(t) + fI_{r,rc}(t)} \tag{SI.34}$$

where, rt was prey abundance before the dynamics within a prey cluster was calculated by using Eq. (SI.44); sI, nI, pI, and fI were the effect of the competition in a prey cluster, the effect of smaller-bodied prey clusters on larger-bodied prey, the effect of larger-bodied prey clusters on smaller-bodied prey, and the effect of fish's foraging for prey, respectively. sI, nI, pI, and fI were calculated by using allometric relationships derived from ecological power laws (Eq. (SI.35), Eq. (SI.38), Eq. (SI.42)), which are described in the following subsections. ri was the intrinsic growth rate of a size class of a prey cluster. ri was 1 for the size classes of the smallest-bodied prey cluster, but it was 0 for the size classes of other prey clusters.

3.1.1. Within-trophic competition and trophic interactions. We used a body-mass-abundance relationship of ecological power laws (Marquet, 2002; Jennings & Mackinson, 2003) (Eq. (SI.36)) to calculate the effect of the competition within a prey cluster on prey abundance, sI, which was given by Eq. (SI.35).

$$sI_r(t) = \frac{1}{ra_r} \sum_{rc} rsc_{r,rc}(t-1) \tag{SI.35}$$

where, rsc was prey abundance after the dynamics within a prey cluster was calculated by using Eq. (SI.44). We used Eq. (SI.36) to describe the body-mass-abundance relationship, and the body-mass-abundance relationship was used to calculate the expected prey abundance, ra.

$$ra_r = cA(rMu_r)^{-eA} \tag{SI.36}$$

where, ra, cA, eA, and rMu were the expected prey cluster abundance, a constant for prey body mass, an exponent for prey body mass, and the representative body mass of a prey cluster, respectively. eA estimated at Tuesday Lake in 1984 (Reuman *et al.* , 2008), Tuesday Lake in 1986 (Jonsson *et al.* , 2005; Reuman *et al.* , 2008), and Ythan Estuary (Reuman *et al.* , 2008) were 0.84, 0.75, and 1.1, respectively. We used a local sensitivity analysis to study the effect of eA on fish morphological evolution. The default value for eA was 0.75.

The strength of trophic interactions was a function of predator-prey body mass ratio (West *et al.* , 1997; Brown & Gillooly, 2003; Emmerson & Raffaelli, 2004; O'Gorman *et al.* , 2010). The effect of the predation by larger-bodied prey clusters on smaller-bodied prey, nI, was described by Eq. (SI.37).

$$nI_{r,rc}(t) = \sum_{\substack{r \\ r \neq r}} \left[fnc((\sum_{rc} rsc_{r,rc}(t-1)), in_{r,r}, \frac{cH_r}{ra_r}, bf_r) \right] \tag{SI.37}$$

where, fnc (Eq. (SI.25)) was a functional response; cH and bf were handling time and an exponent for prey abundance in a functional response, respectively. The default value for cH was 0.1 to reduce the foraging efficiency by 50% when the abundance of prey was 10% of the abundance predicted by the body-mass-abundance relationship (Eq. (SI.36)). in was a scaling factor for nI, and it was a function of predator-prey body mass ratio. We used

Eq. (SI.38) to calculate in.

$$in_{r1,r2} = \begin{cases} ci(\frac{rMu_{r1}}{rMu_{r2}})^{eI} & \text{if } rMu_{r2} \geq rMu_{r1} \\ 0 & \text{if } rMu_{r2} < rMu_{r1} \end{cases} \tag{SI.38}$$

where, *r1* and *r2* were the indices for a smaller-bodied prey cluster and a larger-bodied prey cluster, respectively; ci (default value $= 2.5 \times 10^{-6}$)was a scaling factor for predator-prey body mass ratio; eI was an exponent for predator-prey body mass ratio. The default value for eI was 0.25, which was the theoretically predicted value based on an allometric scaling relationship to approximate basal metabolic rate per unit body mass (West *et al.* , 1997; Brown & Gillooly, 2003; O'Gorman *et al.* , 2010).

Eq. (SI.39) described the effect of smaller-bodied prey clusters on larger-bodied prey, pI.

$$pI_{r,rc}(t) = \sum_{\substack{rk \\ rk \neq r}} \left[ip_{rk,r} \sum_{rc} rsc_{rk,rc}(t-1) \right] \tag{SI.39}$$

where, ip was a scaling factor for the effect of foraging by a smaller-bodied cluster on larger-bodied prey. ip was given by Eq. (SI.38).

$$ip_{r2,r1} = pc \frac{fnc(\sum_{rc} rsc_{r1,rc}(t-1), in_{r1,r2}, \frac{cH_{r1}}{ra_{r1}}, bf_{r1})}{\sum_{rc} rsc_{r1,rc}(t-1)} \tag{SI.40}$$

where, *r1* and *r2* were the indices for a smaller-bodied cluster and a larger-

bodied cluster, respectively; *pc* (default value = 0.01) was a scaling factor for predator-prey body mass ratio; *fnc* was a functional response (Eq. (SI.25)); *cH* was handling time; *bf* was an exponent for prey abundance; *ra* was the prey cluster abundance predicted by the body-mass-abundance relationship (Eq. (SI.36)).

3.1.2. Fish's foraging effect on prey abundance. The effect of alewives's foraging on prey abundance, *fI*, was given by Eq. (SI.41).

$$fI_{r,rc}(t) = \sum [fc_{r,s} nc_{r,rc}(t) cn] \tag{SI.41}$$

$$fc_r = cn\ ci(\frac{rMu_r}{wf})^{eI} \tag{SI.42}$$

The number of size classes in a prey cluster, *cn*, scaled *fI* to set the effect of foraging by an individual on a size class of a prey cluster to *fc* when an individual spent an equal proportion of the total foraging time for all size classes of a prey cluster with the maximum foraging efficiency (= 1). The representative body mass for alewives (wf = 30.27g) was the mean body mass of female alewives of the largest genotype at the stable-stage distribution, which was calculated from a Leslie matrix shown in the fish vital processes section. *nc* was the number of alewives foraging for prey, and it was given by Eq. (SI.27).

$$nc_{r,rc}(t) = \sum_i \left[\frac{frp_{i,r,rc}(t)}{rsc_{r,rc}(t-1)} ft_{i,r,rc}(t) \right] \tag{SI.43}$$

where, *frp* was the efficiency of foraging with a functional response (Eq. (SI.24)); *ft* was foraging time.

3.2. Dynamics within prey cluster

A fraction of prey in a size class of a prey cluster was assumed to move to neighbour size classes for each time step because of genetic mutation. Eq. (SI.44) described this process, and it was calculated after prey abundance, rt, was calculated by using Eq. (SI.34).

$$rsc_{r,rc}(t) = \begin{cases} [(1 - mt_r)rt_{r,rc}(t)] + [\frac{rc-1}{tp_r}mt_r rt_{r,rc-1}(t)] \\ +[(1 - \frac{rc+1}{tp_r})mt_{rc} rt_{r,rc+1}(t)] & \text{if } 1 < rc < tp_r \\ [(1 - mt_r)rt_{r,rc}(t)] + [(1 - \frac{rc+1}{tp_r})mt_r rt_{r,rc+1}(t)] & \text{if } rc = 1 \\ [(1 - mt_r)rt_{r,rc}(t)] + [\frac{rc-1}{tp_r}mt_r rt_{r,rc-1}(t)] & \text{if } rc = tp_r \end{cases} \tag{SI.44}$$

$$mt_r = \binom{tp_r}{1}(mta_r)^1(1 - mta_r)^{tp_r - 1} \tag{SI.45}$$

where, rsc was prey abundance after the dynamics within a prey cluster was calculated by using Eq. (SI.44); rt was prey abundance before the dynamics within a prey cluster was calculated by using Eq. (SI.44); mta was the mutation rate for prey; tp was the number of alleles in the loci determining prey body size +1; mt was the fraction of prey moving to neighbour size classes. For simplicity, we assumed that only one mutation occur for prey for each

time step.

4. Initial condition

Only prey had been simulated for the first 1000 years to form size-structured prey clusters without alewives. At the 1001th year, adult alewives (500 males and 500 females) were introduced into an ecosystem. Under the default parameter setting, when the default values for ci, pc, and cA were used, the abundance of prey clusters converged to positive values when alewives were introduced into the ecosystem. The alleles for the alewives of the initial population were randomly drawn by using allele frequencies. The default value for the initial frequency of the allele improving growth ($P(A)_{t=0}$) was 0.99, and that for the frequency of the allele increasing a gill-raker number ($P(B)_{t=0}$) was 0.01; therefore, we attempted to describe anadromous alewives with standing genetic variation for the initial population. We used a local sensitivity analysis to study the effect of the initial allele frequencies on fish morphological evolution.

We used the body-mass-abundance relationship (Eq. (SI.36)) to set the abundance of organisms in a prey cluster for the initial time step. The initial size distribution for prey was given by Eq. (SI.46).

$$rsc_{r,rc}(0) = ra_r \left[cdfNorm(bl_{r,rc} + \frac{6\, wSD_r}{tp_r}, rMu_r, wSD_r) - cdfNorm(bl_{r,rc}, rMu_r, wSD_r) \right] \tag{SI.46}$$

where, $cdfNorm$ was the cumulative normal distribution function; tp was the number of alleles in the loci determining prey body size +1; rMu was the representative body mass of a prey cluster; wSD (default value $= 0.3 \times rMu$) was the standard deviation of body mass in a prey cluster; bl was the lower bound of a size class (bin).

References

Bagenal, T. B. 1967. A short review of fish fecundity. *The biological basis of freshwater fish production*, 89–111.

Barrowman, N. J. 2000. Nonlinear mixed effects models for meta-analysis. *Ph.D thesis. Dalhousie University, Halifax, Nova Scotia, Canada.*

Beamish, F. W. 1978. Swimming capacity. *Locomotion*, 101.

Beverton, R. J. H., & Holt, S. J. 1957. On the dynamics of exploited fish populations, Fishery Investigations Series II, Vol. XIX, Ministry of Agriculture. *Fisheries and Food*, **1**, 957.

Blaxter, J. H. S., & Fuiman, L. A. 1990. The role of the sensory systems of herring larvae in evading predatory fishes. *Journal of the Marine Biological Association of the United Kingdom*, **70**(02), 413–427.

Brown, J. H., & Gillooly, J. F. 2003. Ecological food webs: high-quality data facilitate theoretical unification. *Proceedings of the National Academy of Sciences*, **100**(4), 1467–1468.

Charnov, E. L. 1976. Optimal foraging, the marginal value theorem. *Theoretical Population Biology*, **9**(2), 129–136.

Drenner, R. W. 1977. The feeding mechanics of the gizzard shad (Dorosoma cepedianum). *University of Kansas.*

Emmerson, M. C., & Raffaelli, D. 2004. Predatorprey body size, interaction strength and the stability of a real food web. *Journal of Animal Ecology*, **73**(3), 399–409.

Folkvord, A., & Hunter, J. R. 1986. Size-specific vulnerability of northern anchovy, Engraulis mordax, larvae to predation by fishes. *Fishery Bulletin*, **84**(4), 859–869.

Friedland, K. D., Ahrenholz, D. W., Smith, J. W., Manning, M., & Ryan, J. 2006. Sieving functional morphology of the gill raker feeding apparatus of Atlantic menhaden. *Journal of Experimental Zoology*, **305**, 974–985.

Gibson, A. J. F., & Myers, R. A. 2003. A statistical, age-structured, life-history-based stock assessment model for anadromous Alosa. *Pages 275–284 of: American Fisheries Society Symposium*. American Fisheries Society.

Gibson, A. J. F., & Myers, R. A. 2004. Estimating reference fishing mortality rates from noisy spawner recruit data. *Canadian Journal of Fisheries and Aquatic Sciences*, **61**(9), 1771–1783.

Gibson, R. N. 1988. Development, morphometry and particle retention capability of the gill rakers in the herring, Clupea harengus L. *Journal of Fish Biology*, **32**(6), 949–962.

Gross, M. R. 1987. Evolution of diadromy in fishes. *Pages 14–25 of: American fisheries society symposium*, vol. 1.

Hubbs, C., & Blaxter, J. H. S. 1986. Ninth larval fish conference: Development of sense organs and behaviour of teleost larvae with special reference to feeding and predator avoidance. *Transactions of the American Fisheries Society*, **115**(1), 98–114.

Janssen, J. 1976. Feeding modes and prey size selection in the alewife (Alosa pseudoharengus). *Journal of the Fisheries Board of Canada*, **33**(9), 1972–1975.

Janssen, J. 1980. Alewives (Alosa pseudoharengus) and ciscoes (Coregonus artedii) as selective and non-selective planktivores. *Evolution and Ecology of Zooplankton Communities*, 580–586.

Jennings, S., & Mackinson, S. 2003. Abundancebody mass relationships in size-structured food webs. *Ecology Letters*, **6**(11), 971–974.

Jonsson, T., Cohen, J. E., & Carpenter, S. R. 2005. Food webs, body size, and species abundance in ecological community description. *Advances in Ecological Research*, **36**, 1–84.

Joseph, E. B., & Davis, J. 1965. A preliminary assessment of the river herring stocks of lower Chesapeake Bay. *Virginia Institute of Marine Science. Species Science Report*, **51**, 1–23.

Juanes, F., Stouder, D. J., & Feller, K. L. 1994. What determines prey size selectivity in piscivorous fishes? *Pages 79–100 of: D.J. Stouder, K.L.*

Fresh, R.J. Feller (Eds.), Theory and Application in Fish Feeding Ecology, vol. 1.

Kahilainen, K. K., Siwertsson, A., Gjelland, K. Ø., Knudsen, R., Bøhn, T., & Amundsen, P. 2011. The role of gill raker number variability in adaptive radiation of coregonid fish. *Evolutionary Ecology*, **25**(3), 573–588.

Keast, A., & Webb, D. 1966. Mouth and body form relative to feeding ecology in the fish fauna of a small lake, Lake Opinicon, Ontario. *Journal of the Fisheries Board of Canada*, **23**(12), 1845–1874.

Kleiber, M. 1932. Body size and metabolism. *ENE*, **1**, E9.

Kohler, C. C., & Ney, J. J. 1980. Piscivority in a land-locked alewife (Alosa pseudoharengus) population. *Canadian Journal of Fisheries and Aquatic Sciences*, **37**(8), 1314–1317.

Lackey, R. T. 1970. Observations on newly introduced landlocked alewives in Maine. *New York Fish and Game Journal*, **17**(2), 110–116.

Langeland, A., & Nst, T. 1995. Gill raker structure and selective predation on zooplankton by particulate feeding fish. *Journal of Fish Biology*, **47**(4), 719–732.

Leslie, P. H. 1958. A stochastic model for studying the properties of certain biological systems by numerical methods. *Biometrika*, 16–31.

Liu, P., & Elaydi, S. N. 2001. Discrete competitive and cooperative models of Lotka-Volterra type. *Journal of Computational Analysis and Applications*, **3**(1), 53–73.

Lorenzen, K. 1996. A simple von Bertalanffy model for density-dependent growth in extensive aquaculture, with an application to common carp (Cyprinus carpio). *Aquaculture*, **142**(3), 191–205.

Lorenzen, K. 2000. Population dynamics and management. *Pages 163–225 of: Tilapias: biology and exploitation.* Springer.

MacNeill, D. B., & Brandt, S. B. 1990. Ontogenetic shifts in gill-raker morphology and predicted prey capture efficiency of the alewife, Alosa pseudoharengus. *Copeia*, 164–171.

Marcy Jr, B. C. 1969. Age determinations from scales of Alosa pseudoharengus (Wilson) and Alosa aestivalis (Mitchill) in Connecticut waters. *Transactions of the American Fisheries Society*, **98**(4), 622–630.

Marquet, P. A. 2002. Of predators, prey, and power laws. *Science*, **295**(5563), 2229–2230.

Mullen, D. M., Fay, C. W., & Moring, J. R. 1986. *In: Alewife, blueback herring.* Fish and Wildlife Service, US Department of the Interior.

Myers, R. A., Bowen, K. G., & Barrowman, N. J. 1999. Maximum reproductive rate of fish at low population sizes. *Canadian Journal of Fisheries and Aquatic Sciences*, **56**(12), 2404–2419.

Myers, R. A., MacKenzie, B. R., Bowen, K. G., & Barrowman, N. J. 2001. What is the carrying capacity for fish in the ocean? A meta-analysis of population dynamics of North Atlantic cod. *Canadian Journal of Fisheries and Aquatic Sciences*, **58**(7), 1464–1476.

Nielsen, L. A. 1980. Effect of walleye (Stizostedion vitreum vitreum) predation on juvenile mortality and recruitment of yellow perch (Perca flavescens) in Oneida Lake, New York. *Canadian Journal of Fisheries and Aquatic Sciences*, **37**(1), 11–19.

O'Gorman, E. J., Jacob, U., Jonsson, T., & Emmerson, M. C. 2010. Interaction strength, food web topology and the relative importance of species in food webs. *Journal of Animal Ecology*, **79**(3), 682–692.

Olalla-Trraga, M. ., Rodrguez, M. ., & Hawkins, B. A. 2006. Broad-scale patterns of body size in squamate reptiles of Europe and North America. *Journal of Biogeography*, **33**(5), 781–793.

ONeill, J. T. 1980. Aspects of the life histories of anadromous alewife and the blueback herring, Margaree River and Lake Ainsle, Nova Scotia, 1978-1979. *Master's thesis. Acadia University, Wolfville, Nova Scotia, Canada.*

Palkovacs, E. P., & Post, D. M. 2008. Eco-evolutionary interactions between predators and prey: can predator-induced changes to prey communities feed back to shape predator foraging traits. *Evolutionary Ecology Research*, **10**, 699–720.

Palkovacs, E. P., Dion, K. B., Post, D. M., & Caccone, A. 2008. Independent evolutionary origins of landlocked alewife populations and rapid parallel evolution of phenotypic traits. *Molecular Ecology*, **17**(2), 582–597.

Persson, L. 1990. Predicting ontogenetic niche shifts in the field: what can be gained by foraging theory? *Pages 303–321 of: Behavioural mechanisms of food selection.* Springer.

Popova, O. A. 1967. The predator-prey relationship among fish. *The biological basis of freshwater fish production. Wiley, New York*, 359–376.

Popova, O. A. 1978. The role of predaceous fish in ecosystems. *Ecology of Freshwater Fish Production*, 215–249.

Reuman, D. C., Mulder, C., Raffaelli, D., & Cohen, J. E. 2008. Three allometric relations of population density to body mass: theoretical integration and empirical tests in 149 food webs. *Ecology Letters*, **11**(11), 1216–1228.

Reznick, D. A., Bryga, H., & Endler, J. A. 1990. Experimentally induced life-history evolution in a natural population. *Nature*, 357–359.

Robinson, B. W., & Parsons, K. J. 2002. Changing times, spaces, and faces: tests and implications of adaptive morphological plasticity in the fishes of northern postglacial lakes. *Canadian Journal of Fisheries and Aquatic Sciences*, **59**(11), 1819–1833.

Rosenzweig, M. L. 1968. The strategy of body size in mammalian carnivores. *American Midland Naturalist*, 299–315.

Sanderson, S. L., Cheer, A. Y., Goodrich, J. S., Graziano, J. D., & Callan, W. T. 2001. Crossflow filtration in suspension-feeding fishes. *Nature*, **412**(6845), 439–441.

Sandlund, O. T., Gjelland, K. ., Bhn, T., Knudsen, R., & Amundsen, P. 2013. Contrasting population and life history responses of a young morph-pair of European whitefish to the invasion of a specialised coregonid competitor, vendace. *PloS one*, **8**(7), e68156.

Scharf, F. S., Juanes, F., & Rountree, R. A. 2000. Predator size-prey size relationships of marine fish predators: interspecific variation and effects of ontogeny and body size on trophic-niche breadth. *Marine Ecology Progress Series*, **208**, 229–248.

Schielke, E. G., Palkovacs, E. P., & Post, D. M. 2011. Eco-evolutionary feedbacks drive niche differentiation in the alewife. *Biological Theory*, **6**(3), 211–219.

Schluter, D., & McPhail, J. D. 1992. Ecological character displacement and speciation in sticklebacks. *American Naturalist*, 85–108.

Schneider, J. C. 2000. *In: Manual of fisheries survey methods II: with periodic updates*. Michigan Department of Natural Resources, Fisheries Division.

Schoener, T. W. 1971. Theory of feeding strategies. *Annual Review of Ecology and Systematics*, 369–404.

Scott, W. B., & Scott, M. G. 1988. *In: Atlantic fishes of Canada.* Published by the University of Toronto Press in cooperation with the Minister of Fisheries and Oceans and the Canadian Government Publishing Centre, Supply and Services Canada.

Shin, Y., & Rochet, M. 1998. A model for the phenotypic plasticity of North Sea herring growth in relation to trophic conditions. *Aquatic Living Resources*, **11**(05), 315–324.

Smith, J. C., & Sanderson, S. L. 2008. Intra-oral flow patterns and speeds in a suspension-feeding fish with gill rakers removed versus intact. *The Biological Bulletin*, **215**(3), 309–318.

Stephens, D. W., & Krebs, J. R. 1986. *In: Foraging theory.* Princeton University Press.

Trexler, J. C. 1989. Phenotypic plasticity in poeciliid life histories. *The Ecology and Evolution of Poeciliid Fishes (Poeciliidae). Prentice Hall, New Jersey*, 201–213.

von Bertalanffy, L. 1960. Principles and theory of growth. *Fundamental aspects of normal and malignant growth*, **493**.

Walters, C. J., & Post, J. R. 1993. Density-dependent growth and competitive asymmetries in size-structured fish populations: a theoretical model and recommendations for field experiments. *Transactions of the American Fisheries Society*, **122**(1), 34–45.

Webb, P. W. 1976. The effect of size on the fast-start performance of rainbow trout Salmo cairdneri, and a consideration of piscivorous predator-prey interactions. *Journal of Experimental Biology*, **65**(1), 157–177.

West, G. B., Brown, J. H., & Enquist, B. J. 1997. A general model for the origin of allometric scaling laws in biology. *Science*, **276**(5309), 122–126.

Supporting information II:

Model input parameter settings

Table SI.2.1: List of symbols

Symbol	Equation([ ]) or algorithm(( ))	Default value	Description
A	[SI.11]	-	The genetic effect on growth
af	[SI.25]	-	Attack rate
B	[SI.20]	-	The genetic effect on a gill-raker number
b	[SI.1]	-	Body length (mm)
bA	[SI.3,SI.10,SI.12]	0.25	The parameter controlling the minimum asymptotic body length
bB	[SI.2,SI.3]	-	The baseline body length growth
bcv	[SI.8]	-	The coefficient variation of body length growth
bf	[SI.25]	-	An exponent for prey density in a functional response
bl	[SI.46]	-	The lower bound for a size class in a prey cluster
bMx	[SI.9,SI.12]	$273.68mm$	The maximum body length

bMn	[SI.12]	-	The minimum body length
bO	[SI.19]	-	Body length (mm) used for calculating the available resource mass for an individual
bR	[SI.12,SI.10]	0.1	The minimum degree of body length development
cA	[SI.35]	5.0×10^{6}	A scaling factor for species body mass-abundance relationship
cD	[SI.16]	10	A scaling factor for $\frac{m}{ir}$ to calculate density-dependent-survival rate
ce	[SI.10,SI.28]	0	The parameter controlling the environmental effect on growth and sexual maturation
cF	[SI.23]	-	The function controlling the foraging efficiency at the midpoint between $log_{10}tu$ and $log_{10}tl$ to 1 when $lp = 0$
cH	[SI.37]	0.1	The parameter controlling handling time
ci	[SI.38,SI.42]	2.5×10^{-6}	A scaling factor for in
cn	[SI.41]	21	The number of size classes for a prey cluster
cp	[SI.18]	-	The number of individuals competing

			for a resource
cV	[SI.9,SI.10]	-	The asymptotic body length
cX	[SI.31]	-	A scaling factor for converting the number of new female offspring to the number of new male and female offspring
ddS	[SI.16]	-	Density-dependent-survival rate
dg	[SI.13]	$log_e 0.75$	A scaling factor for ed
eA	[SI.35]	0.75	An exponent for body-mass-abundance relationship
eag	[SI.29]	Male: 2, Female: 3	The duration (years) that an individual had a chance of sexual maturation -1
eb	[SI.20]	0.125	The effect of body size on fish's foraging for small prey
ed	[SI.10,SI.13,SI.28]	-	The effect of resource mass on body length growth
eI	[SI.38,SI.42]	0.25	An exponent for predator-prey body mass ratio
F	[SI.30]	-	The number of female offspring from a mated female
fc	[SI.42]	-	A scaling factor for the effect

			of foraging by an individual fish on a size class of a prey cluster
fe	[SI.22]	-	Fish foraging efficiency
fI	[SI.41]	-	Fish foraging effect on a resource
fmx	[SI.30]	121.27	The maximum fertility
fnc	[SI.25]	-	Functional response
fp	[SI.31]	-	The proportion of new female offspring
frb	[SI.30]	0.893	An exponent for body mass-fertility conversion
frp	[SI.24]	-	The efficiency of foraging with a functional response
fs	[SI.22]	10	The parameter controlling the difference between the efficiency of foraging on small (or the large) resources and that on medium-sized resources
Fsd	Algorithm 2	$0.25F$	The standard deviation of the number of female offspring from a mated female
ft	[SI.27]	-	The proportion of time an individual foraging a resource

G	algorithm 1	See matrices	The probability an individual surviving and developing to the next life stage
ht	[SI.25]	-	Handling time
i	Index	-	Agent
in	[SI.38]	-	The coefficient for the effect of predators on prey
ip	[SI.40]	-	The coefficient for the effect of prey on predators
ir	[SI.17]	-	The resource mass accessible to an individual for a time step
it	[SI.35]	-	The effect the competition within a prey cluster
kV	[SI.4,SI.9 SI.15,SI.12]	$-log_e(1-0.95)$	An exponent in von Bertalanffy function
l	[SI.20]	-	Gill raker spacing
lp	[SI.21]	0.5	The parameter controlling a decrease in fish foraging efficiency due to small gill-raker spacing
lMx	[SI.19]	$0.2g$	The maximum value for l
lMn	[SI.19]	$0.001g$	The minimum value for l
m	[SI.14]	-	Metabolic rate

mt	[SI.45]	-	The proportion of the resource changing body mass
mta	[SI.45]	1.0×10^{-5}	Mutation rate for resource organisms
μ	[SI.7,SI.6]	-	Mean growth
nc	[SI.43]	-	The number of individuals foraging for a resource
nI	[SI.37]	-	The effect of predators on prey
nll	[SI.20]	8	The number of loci controlling the lower-foraging bound
nlu	[SI.11]	8	The number of loci controlling the upper-foraging bound
nml	[SI.20]	-	The number of alleles lowering the lower-foraging bound
nmu	[SI.11]	-	The number of alleles raising the upper-foraging-size bound
np	[SI.11,SI.20]	2	The number of alleles for a locus
P	Algorithm 1	See matrices in supporting information II	The probability an individual surviving at the current life stage
$P(A)_{t=0}$	-	0.99	The initial frequency of the allele increasing body size
$P(B)_{t=0}$	-	0.01	The initial frequency of the allele

			decreasing gill-raker spacing
pc	[SI.40]	1.0×10^{-2}	A scaling factor for ip
pf	[SI.21]	-	A decrease in the foraging efficacy caused by small gill raker spacing
pI	[SI.39]	-	The effect of prey on predators
r	Index	-	Prey cluster
$r1$	[SI.38,SI.40]	Index	A prey cluster
$r2$	[SI.38,SI.40]	Index	A prey cluster
ra	[SI.36]	-	The expected abundance of a prey cluster
rc	[SI.45]	Index	A size class in a prey cluster
rCV	[SI.33]	0.3	The coefficient variation for the body mass of a prey cluster
ri	[SI.34]	1 for $r = 1$<br>0 for $r > 1$	The intrinsic growth rate for a prey cluster
rMu	[SI.32]	-	The representative body mass of a prey cluster
rMu_1	[SI.32]	$0.01g$	The mean body mass of the prey cluster with the minimum representative body mass
rMu_{rn}	[SI.32]	$5g$	The mean body mass of

			the prey cluster with the maximum representative body mass
rn	[SI.32]	3	The number of prey clusters
rsc	[SI.44,SI.46]	-	The abundance of prey in a size class of a prey cluster after the genetic mutation of prey
rt	[SI.34]	-	The abundance of a resource before mutation
sB	[SI.28]	-	The genetic and environmental effects on sexual maturation
σ	[SI.7,SI.8]	-	standard deviation of growth
sg	Index	-	Life stage
sm	[SI.29]	-	The probability of sexual maturation
t	Index	-	Time
tp	[SI.45]	20	The total number of alleles in the loci determining the body mass) +1
tx	[SI.4,SI.29]	Male: 4<br>Female: 5	The age (year) of the largest morph becoming sexually matured
u	[SI.19]	-	Gape size
w	[SI.30]	-	The body mass (g) of an individual

wf	[SI.41]	30.27g	The representative body mass for alewives
wSD	[SI.33]	-	The standard deviation of the body mass of a prey cluster
x	Index	-	Sex

Table SI.2.2: Parameter values for a local sensitivity analysis about eA, eI, lp, and eb. eA determined with-trophic competitions. eI affected trophic interactions. lp affected the effect of gill-raker spacing on the efficiency of alewives' foraging for large prey. eb determined the effect of fish body size on the efficiency of their foraging for small prey. The number of prey clusters was two or three.

Symbol	Values
eA	0.75, 1, 1.25
eI	0.25, 0.4, 0.55
eb	0, 0.125, 0.25, 0.5, 0.75, 1
lp	0, 0.5, 1

Table SI.2.3: Parameter values for a local sensitivity analysis about the effect size of the alleles for fish growth (bA). eA determined with-trophic competitions. eI affected trophic interactions. eb affected the effect of fish body size on the efficiency of alewives' foraging for small prey. The number of prey clusters was two or three.

Symbol	Values
eA	0.75, 1, 1.25
eI	0.25, 0.4, 0.55
bA	0.125, 0.25, 0.375, 0.5
eb	0, 0.125, 0.25, 0.5

Table SI.2.4: Parameter values for a local sensitivity analysis about the effect of a decrease in the efficiency of their foraging for very large or very small prey (*fs*). *eA* determined with-trophic competitions. *eI* affected trophic interactions. *eb* affected the effect of fish body size on the efficiency of their foraging for small prey. *lp* affected the effect of gill-raker spacing on the efficiency of alewives' foraging for large prey. The number of prey clusters was two or three.

Symbol	Values
eA	0.75, 1 ,1.25
eI	0.25, 0.4, 0.55
eb	0, 0.125, 0.25, 0.5
fs	2.5, 5, 10, 20

Table SI.2.5: Parameter values for a local sensitivity analysis about handling time (cH) in the type II functional response. *eA* determined with-trophic competitions. *eI* affected trophic interactions. *eb* affected the effect of fish body size on the efficiency of alewives' foraging for small prey. The number of prey clusters was two or three.

Symbol	Values
eA	0.75, 1, 1.25
eI	0.25, 0.4, 0.55
cH	0, 0.05, 0.1, 0.25, 0.5
eb	0, 0.125, 0.25, 0.5

Table SI.2.6: Parameter values for a local sensitivity analysis about the individual stochasticity of fish growth (bcv). eA determined with-trophic competitions. eI affected trophic interactions. eb affected the effect of fish body size on the efficiency of alewives' foraging for small prey. The number of prey clusters was two or three.

Symbol	Values
eA	0.75, 1, 1.25
eI	0.25, 0.4, 0.55
bcv	0.001, 0.01, 0.1, 0.2
eb	0, 0.125, 0.25, 0.5

Table SI.2.7: Parameter values for a local sensitivity analysis about the initial allele frequencies ($P(A)_{t=0}$, $P(B)_{t=0}$). $P(A)_{t=0}$ was the initial frequency of the allele improving fish growth. $P(B)_{t=0}$ was the initial frequency of the allele increasing a gill-raker number. eA determined with-trophic competitions. eI affected trophic interactions. The number of prey clusters was two or three.

Symbol	Values
eA	0.75, 1, 1.25
eI	0.25, 0.4, 0.55
$P(A)_{t=0}$	1, 0.9999, 0.999, 0.99, 0.5,<br>0.01, 0.001, 0.0001, 0
$P(B)_{t=0}$	0, 0.0001, 0.001, 0.01, 0.5,<br>0.99, 0.999, 0.9999, 1

Supporting information III:

Figures

Figure SI.3.1: The effect of a decrease in the efficiency of alewives' foraging for very large or very small prey (*fs*) on alewife morphological evolution. A low *fs* value indicated a slow decrease in the efficiency of their foraging for very large or very small prey. $P(\bar{A})$ was the mean frequency of the allele improving their growth. $P(\bar{B})$ was the mean frequency of the allele increasing their gill-raker number. There were three prey clusters. The intensity of within-trophic competitions was low ($eA = 0.75$). Trophic interaction were strong ($eI = 0.25$). A decrease in alewives' body size substantially improved the efficiency of their foraging for small prey ($eb = 0.125$). Alewives' small gill-raker spacing moderately undermine the efficiency of their foraging for large prey ($lp = 0.5$). Allele frequencies were recorded every 150 years in this figure.

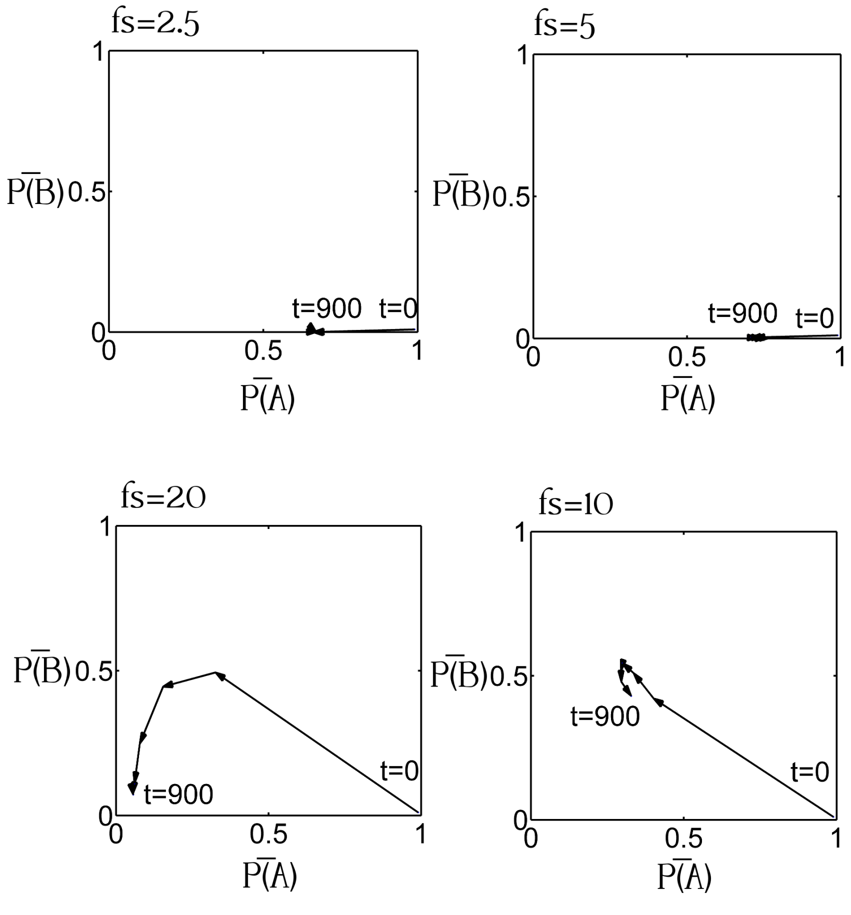

Figure SI.3.2: The effect of handling time (ht) in the type II functional response on alewife morphological evolution. A high ht value indicated long handling time. $P(\bar{A})$ was the mean frequency of the allele improving their growth. $P(\bar{B})$ was the mean frequency of the allele increasing their gill-raker number. There were three prey clusters. The intensity of within-trophic competitions was low ($eA = 0.75$). Trophic interactions were strong ($eI = 0.25$). Body size decrease in alewives substantially improved the efficiency of their foraging for small prey ($eb = 0.125$). Alewives' small gill-raker spacing moderately undermined the efficiency of their foraging for large prey ($lp = 0.5$). Allele frequencies were recorded every 150 years in this figure.

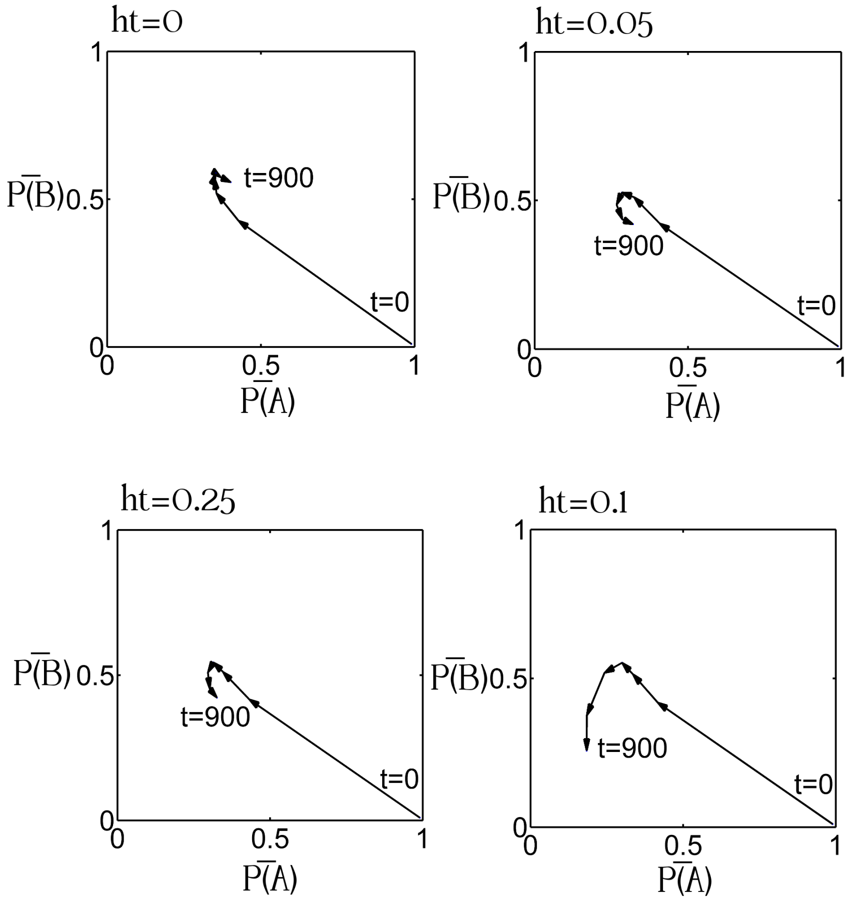

Figure SI.3.3: The effect of the individual stochasticity of alewives' growth (bcv) on alewife morphological evolution. A high bcv value indicated the high individual stochasticity of their growth. $P(\bar{A})$ was the mean frequency of the allele improving their growth. $P(\bar{B})$ was the mean frequency of the allele increasing their gill-raker number. There were three prey clusters. The intensity of within-trophic competitions was low ($eA = 0.75$). Trophic interactions were strong ($eI = 0.25$). A decrease in alewives' body size substantially improved the efficiency of their foraging for small prey ($eb = 0.125$). Alewives' small gill-raker spacing moderately undermined the efficiency of their foraging for large prey ($lp = 0.5$). Allele frequencies were recorded every 150 years in this figure.

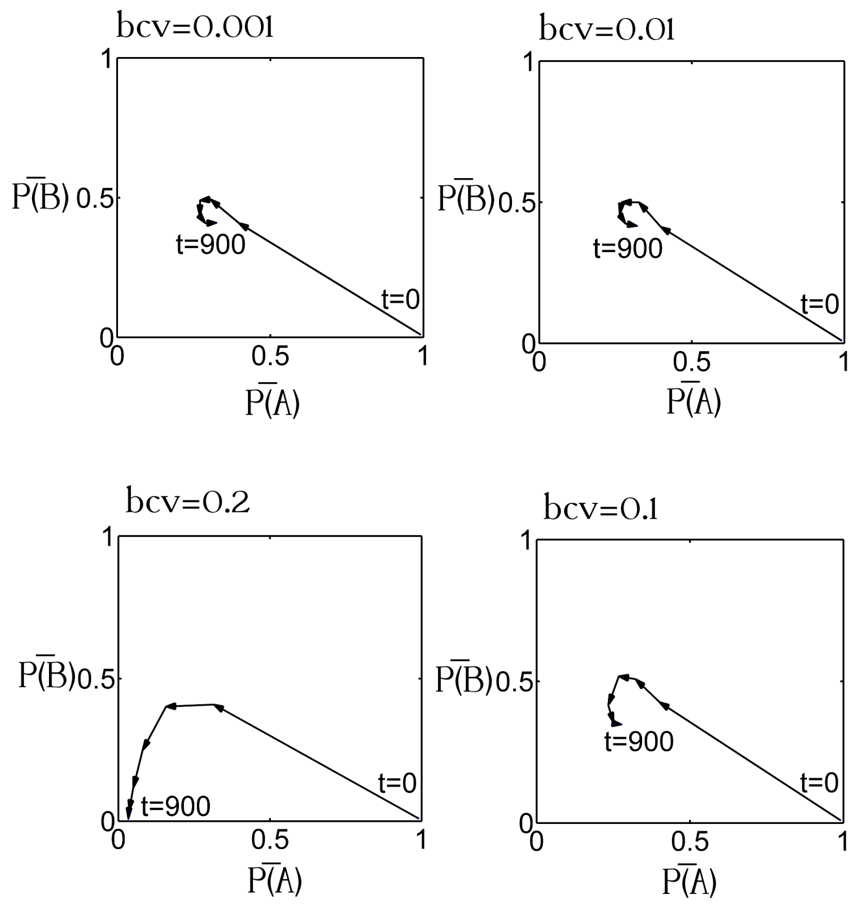

Supplement: Additional file 1 — Contains supporting information I, II, and III. (PDF 833 kb) [file 12862_2017_912_MOESM1_ESM.pdf]
